# Kinetic and inhibition studies on human Jumonji-C (JmjC) domain-containing protein 5†

**DOI:** 10.1039/d2cb00249c

**Published:** 2023-03-20

**Authors:** Anthony Tumber, Eidarus Salah, Lennart Brewitz, Thomas P. Corner, Christopher J. Schofield

**Affiliations:** a Chemistry Research Laboratory, Department of Chemistry and the Ineos Oxford Institute for Antimicrobial Research, University of Oxford 12 Mansfield Road OX1 3TA Oxford UK christopher.schofield@chem.ox.ac.uk lennart.brewitz@chem.ox.ac.uk

## Abstract

Jumonji-C (JmjC) domain-containing protein 5 (JMJD5) is a human 2-oxoglutarate (2OG) and Fe(ii)-dependent oxygenase which catalyses the post-translational C3 hydroxylation of arginyl-residues and which is linked to the circadian rhythm and to cancer biology through as yet unidentified mechanisms. We report robust solid phase extraction coupled to mass spectrometry (SPE-MS)-based JMJD5 assays which enable kinetic and high-throughput inhibition studies. The kinetic studies reveal that some synthetic 2OG derivatives, notably including a 2OG derivative with a cyclic carbon backbone (*i.e.* (1*R*)-3-(carboxycarbonyl)cyclopentane-1-carboxylic acid), are efficient alternative cosubstrates of JMJD5 and of factor inhibiting hypoxia-inducible transcription factor HIF-α (FIH), but not of the Jumonji-C (JmjC) histone *N*^ε^-methyl lysine demethylase KDM4E, apparently reflecting the closer structural similarity of JMJD5 and FIH. The JMJD5 inhibition assays were validated by investigating the effect of reported 2OG oxygenase inhibitors on JMJD5 catalysis; the results reveal that broad-spectrum 2OG oxygenase inhibitors are also efficient JMJD5 inhibitors (*e.g. N*-oxalylglycine, pyridine-2,4-dicarboxylic acid, ebselen) whereas most 2OG oxygenase inhibitors that are in clinical use (*e.g.* roxadustat) do not inhibit JMJD5. The SPE-MS assays will help enable the development of efficient and selective JMJD5 inhibitors for investigating the biochemical functions of JMJD5 in cellular studies.

## Abstract 2

Robust mass spectrometry-based assays are reported which monitor catalysis of the 2-oxoglutarate-dependent oxygenase Jumonji-C domain-containing protein 5 (JMJD5). The assays have been applied to both kinetic and inhibition studies.

## Graphical abstract

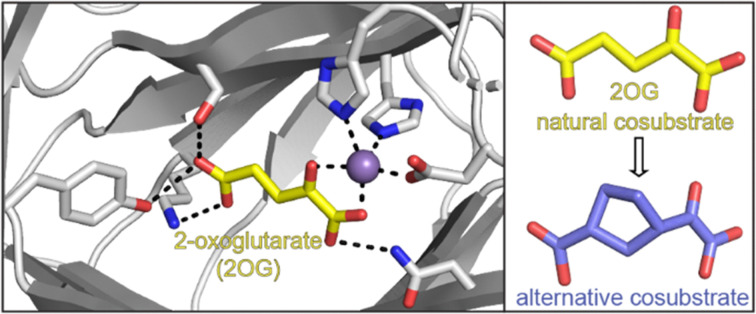

## Introduction

Approximately 60–70 human 2-oxoglutarate (2OG) and Fe(ii)-dependent oxygenases have been identified, some of which have important biological functions, including in DNA/RNA damage repair,^1,2^ hypoxia signalling,^3,4^ extracellular matrix biosynthesis,^4,5^ lipid and small-molecule metabolism,^6,7^ and histone/chromatin modification.^8,9^ However, several predicted human 2OG oxygenases and structurally related proteins have not yet been assigned biochemical functions, *e.g.* PHD finger protein 2 (PHF2),^10^ phytanoyl-CoA dioxygenase domain-containing protein 1 (PHYD1),^11^ and aspartate β-hydroxylase domain-containing proteins 1 and 2 (AspHD1 and AspHD2),^12,13^ while others have been assigned apparently contradictory biochemical functions, *e.g.* Jumonji-C (JmjC) domain-containing protein 6 (JMJD6)^14,15^ and JmjC domain-containing protein 5 (JMJD5).^16–25^

**Fig. 1:**
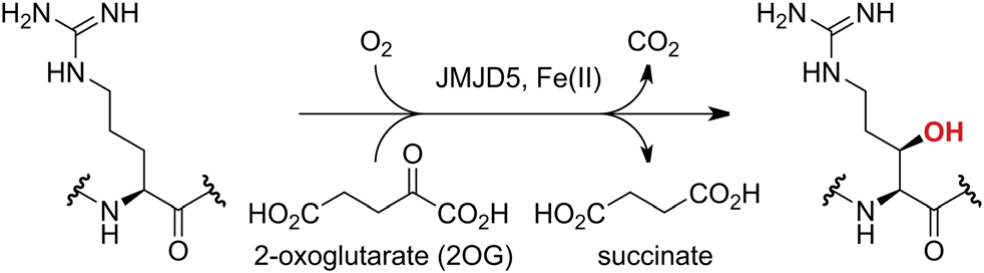
JMJD5 catalyses the stereospecific C3 hydroxylation of arginyl residues.^32^

JMJD5 is essential during embryogenesis and is reported to be involved in circadian rhythm as well as in cancer progression/suppression.^26–31^ Initially, JMJD5 was assigned as a JmjC histone *N*^ε^-methyl lysine demethylase (*i.e.* KDM8)^16–18^ and, later, as a protease that catalyses the hydrolysis of histone tails.^19–21^ However, the cellular evidence of demethylase activity for JMJD5 has not been reproduced in studies with isolated JMJD5 and in cellular studies.^22–25^ By contrast, we have reported matrix-assisted laser desorption/ionization time-of-flight (MALDI-TOF) mass spectrometry (MS)-based assays showing that isolated recombinant human JMJD5 catalyses the stereospecific C3 hydroxylation of arginyl-residues in fragments of RCC1 domain containing 1 (RCCD1) and the 40S ribosomal protein S6 (RPS6), but not the demethylation of the tested histone *N*^ε^-methyl lysine residues (Fig. 1).^32^ Although JMJD5 may have other substrates, these observations are supported by cellular studies which have shown that JMJD5 interacts with RCCD1^33,34^ and by crystallographic studies which have shown that the JMJD5 structure is more similar to human 2OG-dependent protein hydroxylases (*i.e.* oxygenases forming stable alcohol products), *e.g.* factor inhibiting hypoxia-inducible transcription factor HIF-α (FIH), rather than to human JmjC KDMs.^24,25^

Here we describe a robust solid phase extraction coupled to MS (SPE-MS)-based JMJD5 high-throughput assay which enables kinetic studies and which we employed to investigate small molecules acting as JMJD5 cosubstrates and/or inhibitors. The SPE-MS assay will likely facilitate the development of efficient and selective JMJD5 small-molecule inhibitors for cellular functional assignment studies.

## Results and discussion

### Development of a SPE-MS JMJD5 assay

**Fig. 2:**
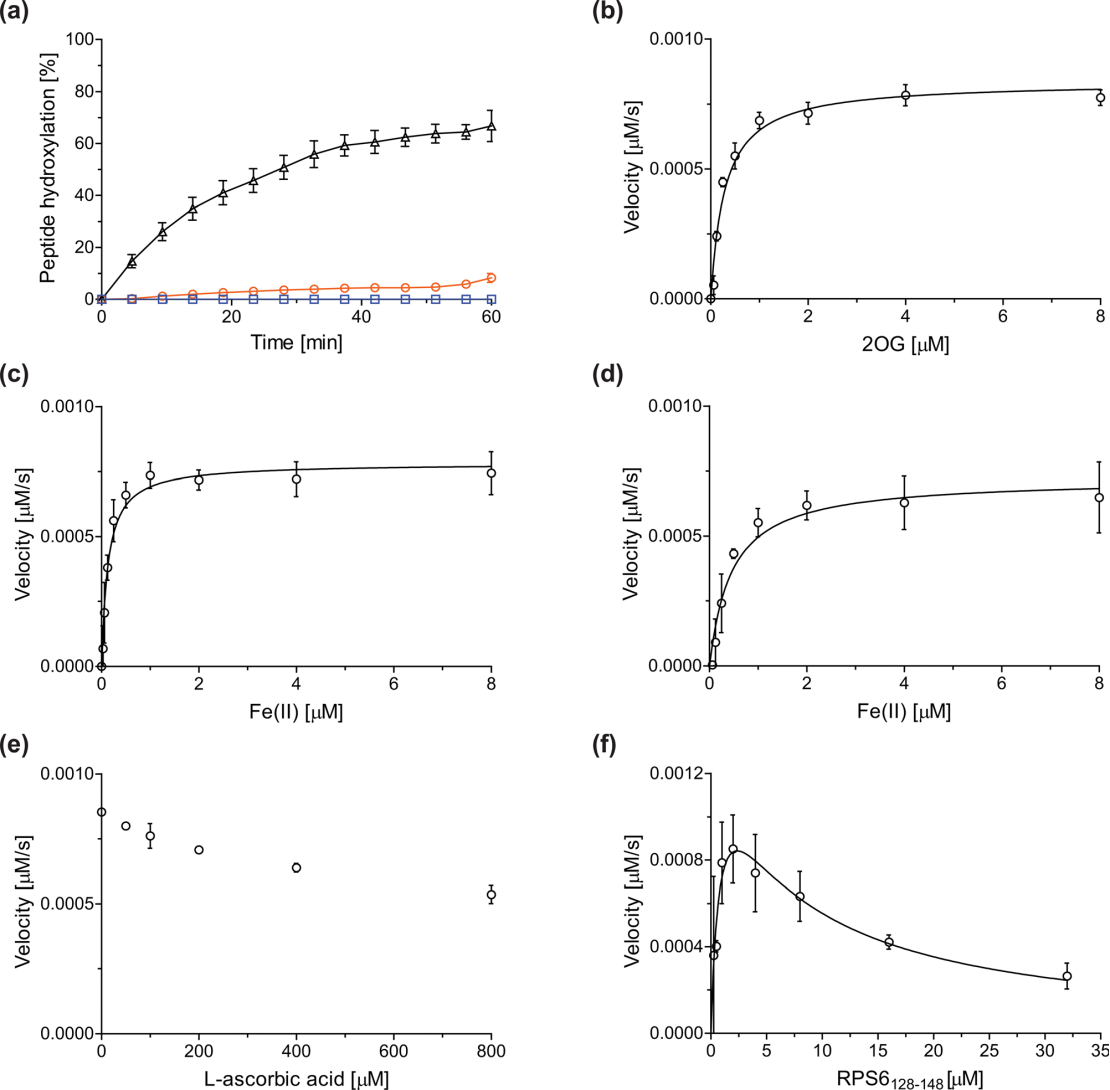
Determination of steady-state kinetic parameters for the JMJD5-catalyzed hydroxylation of RPS6_128–148_ using SPE-MS. (a) Time-course data of the JMJD5-catalyzed hydroxylation of RPS6_128–148_ (black triangles), RPS6_128–148_ (no enzyme control, blue boxes), and RCCD1_134–150_ (orange circles) in buffer (50 mM MOPS, pH 7.5, 20 °C); (b–f) determination of the JMJD5 *v*^app^_max_ and *K*^app^_m_ values for (b) 2OG, (c) Fe(ii) in the presence of l-ascorbic acid (LAA), (d) Fe(ii) in the absence of LAA, (e) LAA, and (f) RPS6_128–148_. Assays employed 0.15 μM JMJD5; details are described in the Experimental section. The initial hydroxylation rates used to determine kinetic parameters are shown in Fig. S1 (ESI†). The results are means of three independent runs (*n* = 3; mean ± standard deviation, SD).

Previous assays for the C3 arginyl-residue hydroxylation of isolated JMJD5 have employed low-throughput NMR and MALDI-TOF MS.^32,35^ To develop an improved JMJD5 high-throughput assay, we investigated SPE-MS which has previously been used to assay 2OG oxygenases. RCCD1- and RPS6-derived oligopeptides suitable for developing SPE-MS JMJD5 assays, which monitor the +16 Da mass shift associated with substrate hydroxylation, were synthesized using solid phase peptide synthesis (SPPS), *i.e.* RCCD1_134–150_ and RPS6_128–148_. Initial SPE-MS turnover assays using RCCD1_134–150_, RPS6_128–148_, and recombinant isolated human full-length JMJD5 revealed that the hydroxylation of RPS6_128–148_ proceeds more efficiently than that of RCCD1_134–150_. Under the optimized conditions (50 mM MOPS, pH 7.5, 20 °C), JMJD5-catalyzed hydroxylation of RPS6_128–148_ reached ∼65% after 1 h incubation, whereas only <10% hydroxylation of RCCD1_134–150_ was observed after the same time (Fig. 2a). Note that the SPE-MS JMJD5 assay uses lower enzyme and substrate concentrations than the reported MALDI-TOF MS assay (0.15 μM respectively 2.0 μM for SPE-MS assays compared to 10 μM respectively 100 μM for MALDI-TOF MS assays).^32,35^ Comparison with a no-enzyme control indicates that JMJD5 catalyses the hydroxylation of RPS6_128–148_, *i.e.* no evidence for a +16 Da mass shift was observed in the control (Fig. 2a).

### Kinetic studies

The levels of JMJD5-catalyzed RPS6_128–148_ hydroxylation, as observed in the SPE-MS assay, were sufficient for kinetic studies. Thus, maximum velocities (*v*^app^_max_) and Michaelis constants (*K*^app^_m_) of JMJD5 were determined for 2OG, Fe(ii), l-ascorbic acid (LAA), and RPS6_128–148_ using SPE-MS assays (Fig. 2 and Table 1); these values were then used to calculate turnover numbers (catalytic constants, *k*^app^_cat_) and specificity constants (*k*_cat_/*K*_m_).

**Table 1:** Steady-state kinetic parameters of JMJD5 determined using SPE-MS^a^

	(Co-)substrate/cofactor	*v* ^app^_max_ [nM s^−1^]	*k* ^app^_cat_ b [s^−1^]	*K* ^app^_m_ [μM]	*k*_cat_/*K*_m_ [mM^−1^ s^−1^]
i	2OG	0.84 ± 0.03	5.6 × 10^−3^ ± 0.2 × 10^−3^	0.29 ± 0.04	19.3 ± 5.8
ii	Fe(ii)^c^	0.75 ± 0.03	5.0 × 10^−3^ ± 0.2 × 10^−3^	0.13 ± 0.02	38.5 ± 6.2
iii	Fe(ii)^d^	0.73 ± 0.05	4.9 × 10^−3^ ± 0.4 × 10^−3^	0.46 ± 0.11	10.7 ± 2.7
iv	RPS6_128–148_^e^	1.5 ± 0.4	10 × 10^−3^ ± 2.7 × 10^−3^	0.87 ± 0.46	11.5 ± 6.3

a Determined using 0.15 μM JMJD5 in buffer (50 mM MOPS, pH 7.5, 20 °C), as described in the Experimental section. The results are means of three independent runs (*n* = 3; mean ± SD).

b *k* ^app^_cat_ values were calculated from *v*^app^_max_ values assuming that the concentration of active JMJD5 equals the total enzyme concentration (note that efficient covalent or tight-binding JMJD5 inhibitors suitable for active site titrations have not yet been described).

c Determined in the presence of 100 μM LAA.

d Determined in the absence of LAA.

e *v*_max_, *k*_cat_, and *K*_m_ values were determined.

The JMJD5 *k*^app^_cat_ values and errors for 2OG and Fe(ii) are similar, within experimental error, in accord with the high robustness and reproducibility of the SPE-MS assay. The JMJD5 *K*^app^_m_ values for 2OG and Fe(ii) are <0.5 μM, suggesting a high affinity of JMJD5 for both of them, consistent with prior studies using MALDI-TOF MS.^32^ In part, this observation may reflect the reported results that the JMJD5-catalysed substrate oxidation-uncoupled oxidative decarboxylation of 2OG to give succinate and CO_2_ is slow.^32^ The *K*^app^_m_ for Fe(ii) is lower in the presence of LAA than in its absence, which may indicate a potential function of LAA in preventing Fe(ii) from being oxidized (Table 1, entries ii and iii).^36^

The SPE-MS *K*^app^_m_ values for Fe(ii) and 2OG are approximately an order of magnitude lower than those obtained using MALDI-TOF MS (∼2.7 μM for Fe(ii), ∼9.5 μM for 2OG^32^), an observation which likely, at least in part, reflects the different assay conditions of SPE-MS assays (0.15 μM JMJD5 in 50 mM MOPS, pH 7.5, 20 °C) and MALDI-TOF MS assays (10.0 μM JMJD5 in 50 mM HEPES, pH 7.5^32^). Note that similar differences between the results of SPE-MS and MALDI-TOF MS assays have been described for other 2OG oxygenases, including for FIH.^37,38^

The kinetic studies indicate that l-ascorbic acid (LAA) is not a cosubstrate/cofactor of isolated recombinant human JMJD5 (Fig. 2e), in accord with similar observations reported for some other human 2OG oxygenases, *e.g.* aspartate/asparagine-β-hydroxylase (AspH).^39^ Nonetheless, JMJD5 SPE-MS assays were performed in the presence of LAA because it improves assay robustness as, *e.g.*, the smaller standard deviation for the Fe(ii) kinetic parameters determined in the presence of LAA manifests (Table 1, entry ii).

To obtain kinetic parameters of JMJD5 for RPS6_128–148_, the data were fitted using non-linear regression to an equation which accounts for substrate inhibition (*Y* = *v*^app^_max_·*X*/(*K*^app^_m_ + *X*·(1 + *X*/*K*_*i*_))), as increased RPS6_128–148_ concentrations appear to impair JMJD5 catalysis (Fig. 2f). The results reveal that RPS6_128–148_ is an efficient JMJD5 substrate, albeit within a narrow concentration range (Table 1, entry iv). Thus, the results inform on ideal RPS6_128–148_ concentrations to be used in JMJD5 turnover and inhibition assays. Note that our previously reported JMJD5 MALDI-TOF MS assays employ a truncated version of the RPS6_128–148_ substrate peptide, *i.e.* RPS6_129–144_,^32^ which, however, was not SPE-MS compatible, a difference which may account for differences in the kinetic parameters of the MS assays. The MALDI-TOF MS assay results did not indicate that RPS6_129–144_ inhibits JMJD5 at high concentrations; however, the obtained *K*_m_ value for RPS6_129–144_ was high compared to that obtained for RPS6_128–148_ using SPE-MS assays and to those reported for other 2OG oxygenases (∼60 μM), potentially also reflecting the different reaction conditions used.^32^

It appears that the JMJD5 *k*^app^_cat_ values are ∼10- to ∼100-fold lower than those reported for other human 2OG oxygenases in studies using SPE-MS assays, *i.e.* FIH, AspH, and KDM4C (Table 2). The JMJD5 *K*^app^_m_ values for 2OG, Fe(ii), and substrate are at the lower end of the range of those reported for human FIH, AspH, and KDM4C using SPE-MS assays, indicating a relatively high affinity of JMJD5 for its (co)substrates/cofactors.^32,35^ While SPE-MS assays have not yet been used to determine kinetic parameters of isolated recombinant HIF-α prolyl hydroxylase domain-containing protein 2 (PHD2), which, like FIH, is of importance in hypoxia sensing and which is a validated medicinal chemistry target,^3,4^ a PHD2 Förster resonance energy transfer (FRET) assay has been employed to determine *K*^app^_m_ values for 2OG, Fe(ii), and substrate under conditions similar to those of SPE-MS assays;^40^ the PHD2 *K*^app^_m_ values for 2OG and Fe(ii) are similar to those of JMJD5, whereas the PHD2 *K*^app^_m_ value for substrate appears to be higher than that of JMJD5. In general, the JMJD5 *k*_cat_/*K*_m_ values are in the range of those reported for FIH and KDM4C, while those reported for AspH appear to be ∼3- to ∼18-fold higher (Table 2).

**Table 2:** Steady-state kinetic parameters of selected 2OG-dependent protein oxygenases

(Co-)substrate/cofactor		JMJD5^a^	FIH^37^ b	AspH^39^ c	KDM4C^41^ d	PHD2^40^ e
2OG	*k*_cat_ [s^−1^]	5.6 × 10^−3^ ± 0.2 × 10^−3^	0.04 ± 0.01	0.19 ± 0.03	0.075 ± 0.001	Not reported
	*K*_m_ [μM]	0.29 ± 0.04	0.8 ± 0.1	0.60 ± 0.09	2.6 ± 0.1	0.35 ± 0.03
	*k*_cat_/*K*_m_ [mM^−1^ s^−1^]	19.3 ± 5.8	47.6 ± 12.5	320 ± 70	28.5 ± 1.3	Not reported

Fe(ii)^f^	*k*_cat_ [s^−1^]	5.0 × 10^−3^ ± 0.2 × 10^−3^	Not reported	0.19 ± 0.03	Not reported	Not reported
	*K*_m_ [μM]	0.13 ± 0.02	Not reported	1.42 ± 0.16	Not reported	0.89 ± 0.07
	*k*_cat_/*K*_m_ [mM^−1^ s^−1^]	38.5 ± 6.2	Not reported	130 ± 30	Not reported	Not reported

Substrate	*k*_cat_ [s^−1^]	10 × 10^−3^ ± 2.7 × 10^−3^	Not reported	0.20 ± 0.03	0.089 ± 0.004	Not reported
	*K*_m_ [μM]	0.87 ± 0.46	Not reported	1.19 ± 0.26	5.8 ± 0.7	7.3 ± 1.3
	*k*_cat_/*K*_m_ [mM^−1^ s^−1^]	11.5 ± 6.3	Not reported	170 ± 50	15.4 ± 1.9	Not reported

a Using JMJD5 (0.15 μM) and RPS6_128–148_ as the substrate.

b Using FIH (0.15 μM) and HIF-1α_788–822_^42^ as the substrate.^37^

c Using AspH_315–758_ (0.1 μM) and a synthetic cyclic peptide (hFX–CP_101–119_)^43^ as the substrate.^39^

d Using KDM4C (0.5 μM) and ARTAQTARK(me3)STGGIA (a histone 3 K9(me3) derivative) as the substrate.^41^

e Using PHD2 (1.0 nM) and HIF-1α-derived biotin-DLEMLAPYIPMDDDFQ as the substrate.^40^

f Determined in the presence of LAA.

### Alternative JMJD5 cosubstrates

Biochemical and crystallographic evidence has been reported that both isolated recombinant human FIH and AspH can accept synthetic and natural 2OG derivatives as cosubstrates to enable substrate hydroxylation in the absence of 2OG; the 2OG oxygenase-catalysed oxidative decarboxylation of the 2OG derivatives to give the corresponding succinate derivatives and CO_2_ was shown to be coupled to substrate peptide hydroxylation.^37,44^ It was thus of interest to investigate whether the reported structural similarities of JMJD5 and FIH^24,25^ manifest in a similar reactivity with 2OG derivatives and whether the reactivity of JMJD5 with 2OG derivatives is different to that of JmjC KDMs. Hence, the ability of synthetic 2OG derivatives to sustain catalysis by isolated recombinant JMJD5 and KDM4E, a representative JmjC KDM, was tested in the absence of 2OG.

JMJD5 was incubated with Fe(ii), LAA, RPS6_128–148_, and 34 synthetic 2OG derivatives, the latter in a relatively high concentration (400 μM) to facilitate substrate turnover in the absence of 2OG; RPS6_128–148_ hydroxylation was monitored using SPE-MS (Table S1, ESI†). The results reveal that of the 34 tested synthetic 2OG derivatives investigated for JMJD5 cosubstrate activity, only one sustains RPS6_128–148_ hydroxylation with similar efficiency as 2OG, *i.e.* (1*R*)-3-(carboxycarbonyl)cyclopentane-1-carboxylic acid (14; Table 3, entry xv). Five others show reduced levels of RPS6_128–148_ hydroxylation with respect to 2OG, *i.e.*1, 6, 8, 10, and 11 (Table 3). For all the tested remaining 2OG derivatives, no substantial levels of JMJD5-catalysed RPS6_128–148_ hydroxylation were detected (Table S1, ESI†). It thus appears that JMJD5 prefers C4-substituted 2OG derivatives as cosubstrates over the C3 isomers, *e.g.* 4-ethyl-2OG (10) and 4-propyl-2OG (11) show weak cosubstrate activity with JMJD5 while the isomeric 3-ethyl-2OG (2) and 3-propyl-2OG (3) do not; a notable exception is the cosubstrate activity of 3-(4-methoxybenzyl)-2OG (6) (Table 3).

**Table 3:** The effect of representative 2OG derivatives on isolated recombinant human JMJD5, FIH, AspH, and KDM4E determined using SPE-MS (results with all the tested 34 2OG derivatives are shown in Table S1)

	2OG derivative^a^	JMJD5^b^ (%)	FIH^37^ c (%)	AspH^44^ d (%)	KDM4E^e^ (%)		2OG derivative^a^	JMJD5^b^ (%)	FIH^37^ c (%)	AspH^44^ d (%)	KDM4E^e^ (%)
i	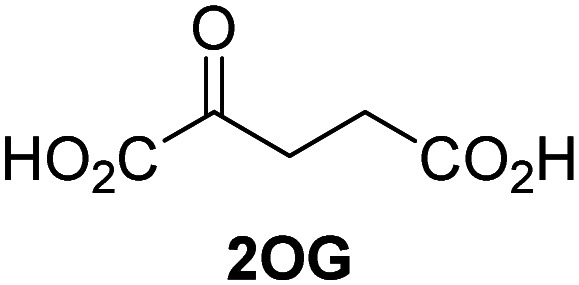	∼40	∼50	>95	∼70	x	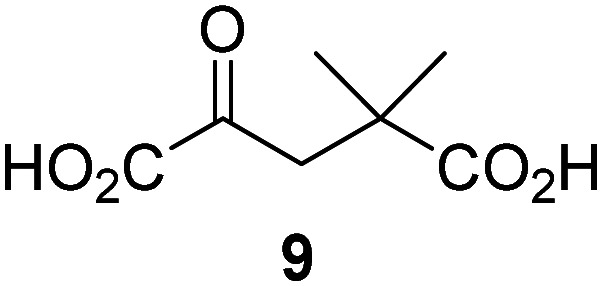	<1	<1	<1	<1
ii	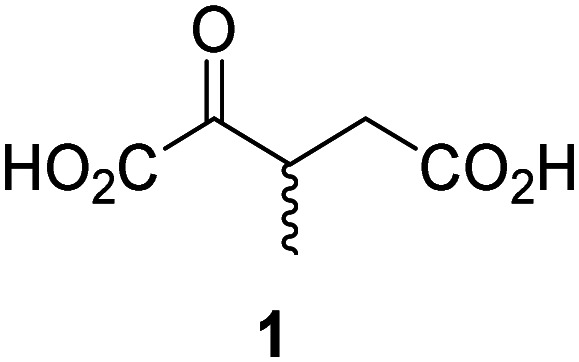	∼10	∼55	>95	∼10	xi	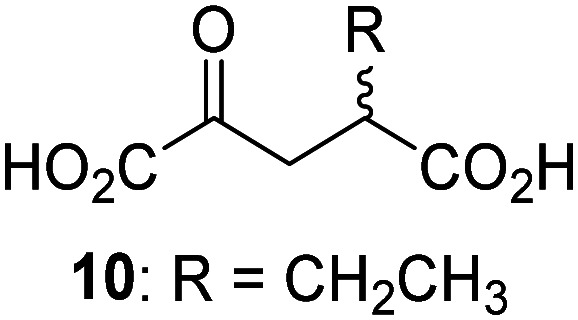	∼10	∼10	<1	<1
iii	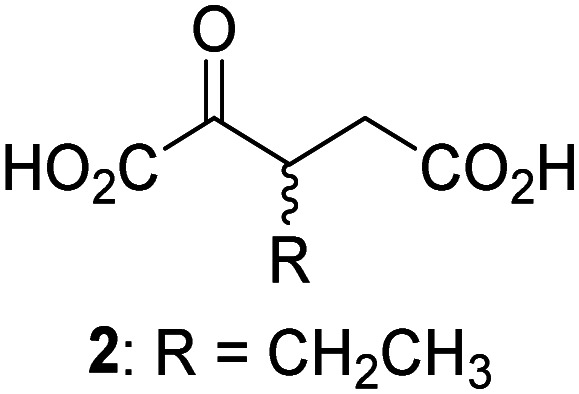	<1	∼2	<1	<1	xii	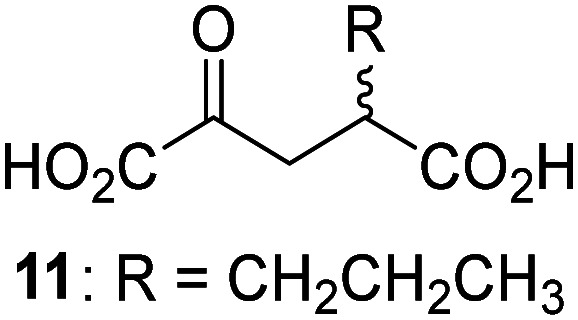	∼10	∼5	<1	<1
iv	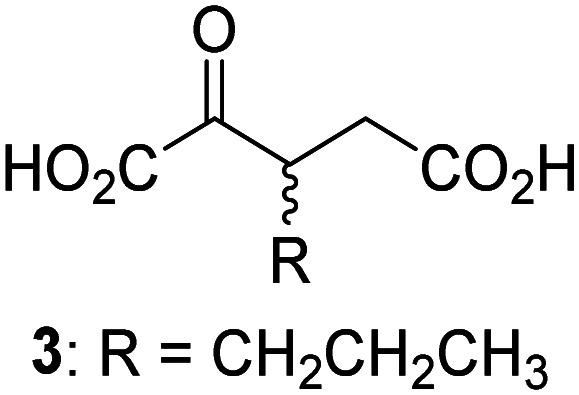	<1	<1	<1	<1	xiii	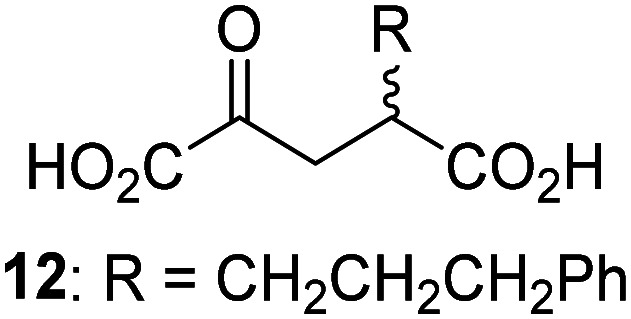	<1	<1	<1	<1
v	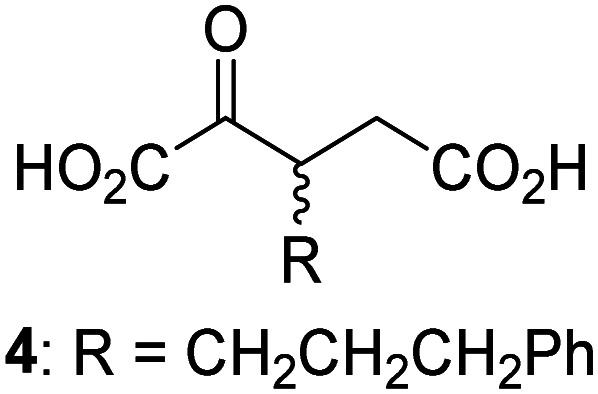	<1	<1	<1	∼2	xiv	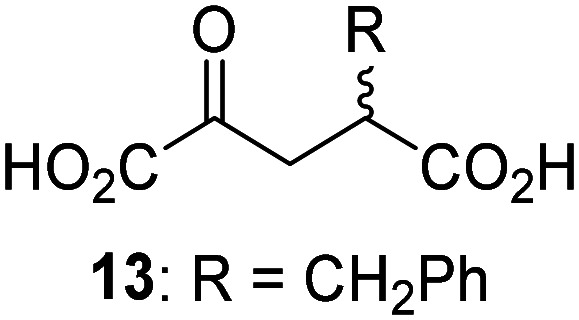	<1	<1	<1	<1
vi	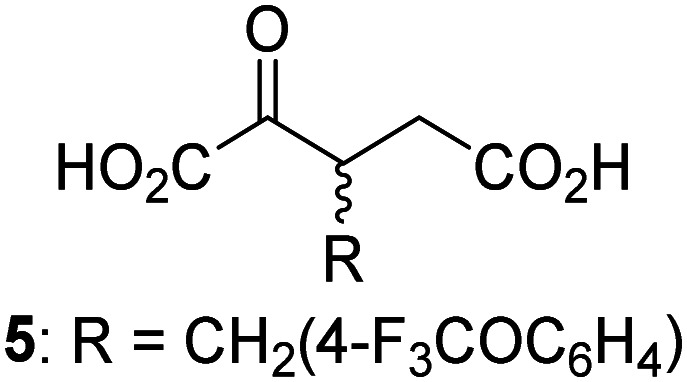	<1	<1	<1	<1	xv^f^	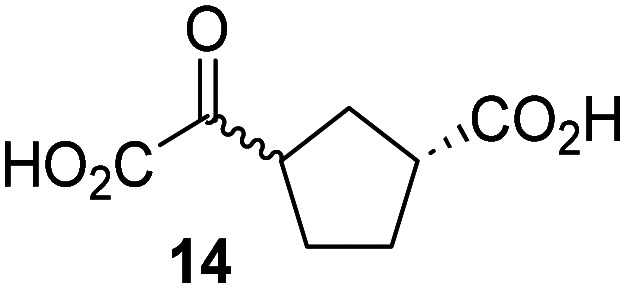	∼35	∼2	∼15	<1
vii	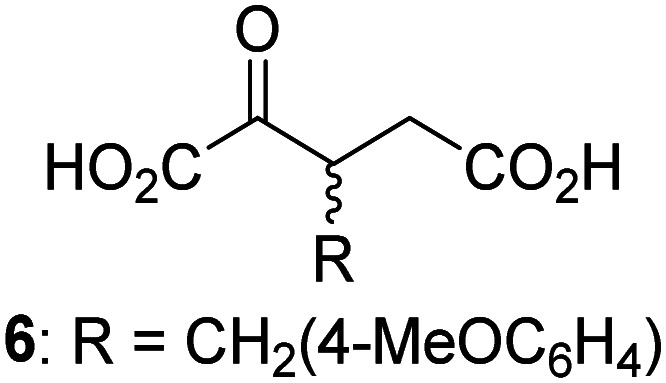	∼10	<1	<1	∼5	xvi	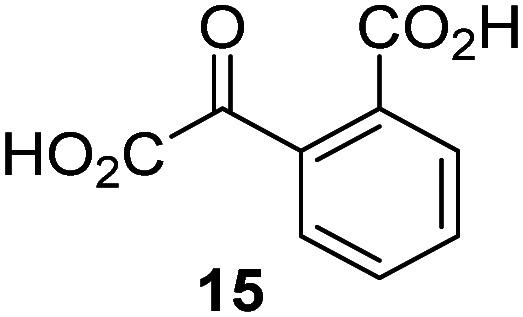	<1	<1	<1	<1
viii	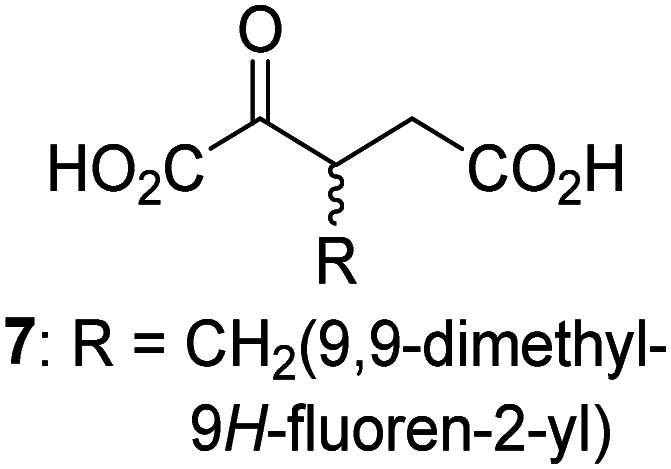	<1	<1	<1	<1	xvii	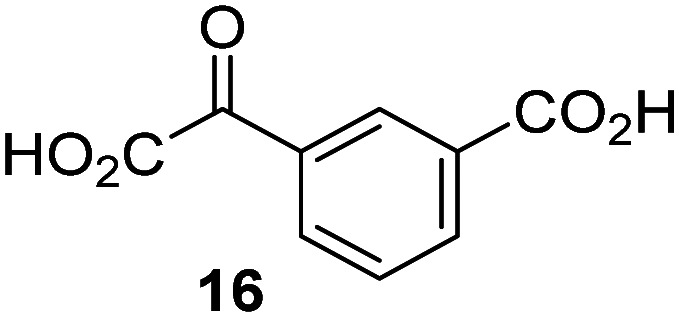	<1	<1	<1	<1
ix	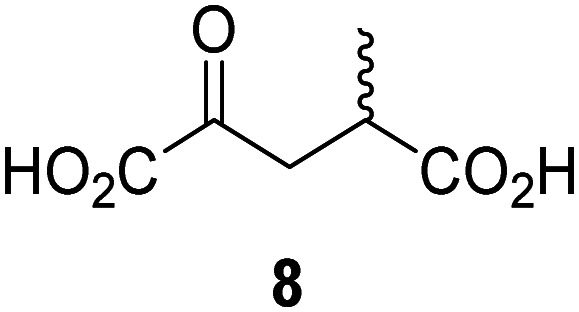	∼10	∼45	∼10	<1	xviii	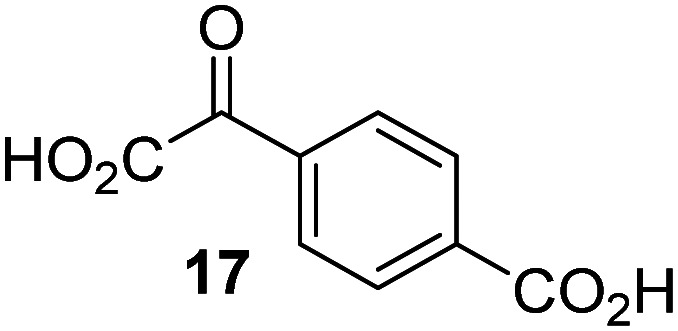	<1	<1	∼80	<1

a 2OG derivatives were prepared as reported,^37,44^ chiral 2OG derivatives were used as racemic mixtures unless indicated otherwise.

b JMJD5 (0.15 μM), 2OG derivative (400 μM), Fe(ii) (20 μM), and RPS6_128–148_ (2.0 μM) in buffer (50 mM MOPS, pH 7.5).

c FIH (0.15 μM), 2OG derivative (330 μM), Fe(ii) (50 μM), and HIF-1α_788–822_^42^ (5.0 μM) in buffer (50 mM Tris, 50 mM NaCl, pH 7.5).^37^

d AspH (0.1 μM), 2OG derivative (330 μM), Fe(ii) (50 μM), and hFX-CP_101–119_ (2.0 μM) in buffer (50 mM HEPES, pH 7.5).^44^

e KDM4E (0.15 μM), 2OG derivative (330 μM), Fe(ii) (50 μM), and ARTAQTARK(me3)STGGIA (a histone 3 K9(me3) derivative)^41^ (10.0 μM) in buffer (50 mM MES, pH 7.0).

f Mixture of diastereomers, dr (*cis* : *trans*) = 1 : 1.

The tested 2OG derivatives appear to be less able to bind to, and react with JMJD5 than with AspH,^44^*i.e.* only 6 of the 34 tested 2OG derivatives were cosubstrates of JMJD5 whereas 11 2OG derivatives are cosubstrates of AspH^44^ (Table 3 and Table S1, ESI†). This observation may reflect the crystallographic observation that the side chains of JMJD5 Trp310, Leu329, and Val402 form a tight hydrophobic pocket around the 2OG ethylene unit, resulting in a smaller 2OG binding pocket and potentially in a reduced ability to accommodate sterically bulky substituents at the 2OG C3 and C4 positions than that of the AspH 2OG binding pocket;^32,35^ note, however, that the JMJD5 and AspH assay conditions differ slightly.

Interestingly, JMJD5 and FIH^37^ appear to accept a structurally similar set of 2OG derivatives as cosubstrates, *i.e.* 2OG derivatives 1, 8, 10, 11, and 14 (Table 3); 2OG derivative 6 is selective as a cosubstrate for JMJD5 over FIH while 2OG derivative 2 is selective as a cosubstrate for FIH (albeit poorly) over JMJD5. These observations are, in general, in accord with the reported structural similarities of the JMJD5 and FIH active sites.^24,25^ The reactivity of FIH and JMJD5 to react with 4-ethyl-2OG (10) and 4-propyl-2OG (11) is particularly notable, because it distinguishes these two 2OG oxygenases from AspH,^44^ which, unlike FIH and JMJD5, is not a JmjC subfamily 2OG oxygenase.^43^ Note, however, that the ability of 2OG oxygenases to react with particular 2OG derivatives might be influenced by other factors than the structure of the 2OG derivative and of the 2OG binding pocket, including *e.g.* by the reaction conditions and interactions involving the substrate.

Notably, of the 34 synthetic 2OG derivatives investigated for JMJD5 cosubstrate activity (Table S1, ESI†), only three were able to sustain the demethylase activity of the human JmjC demethylase KDM4E in the absence of 2OG, *i.e.*1, 4, and 6 (Table 3, entries ii, v, and vii), albeit at reduced levels compared to 2OG. This observation is unexpected considering the apparently relatively large volume of the 2OG binding pockets in the KDM4 subfamily JmjC KDMs.^45,46^ The results reveal that even the most efficient alternative KDM4E cosubstrate identified from the tested set of 2OG derivatives, *i.e.* 3-methyl-2OG (1), is still ∼7-fold less efficient than 2OG whereas at least one of the tested 2OG derivatives displays similar cosubstrate activity with JMJD5, FIH, and AspH as 2OG. Importantly, 2OG derivative 14, which contains a rigid cyclic carbon backbone, was not an efficient KDM4E cosubstrate, consistent with the proposal that the 2OG binding site of JMJD5 resembles that of KDM4E to a lesser extent than that of FIH, in accord with the structural similarities of JMJD5 and FIH^24,25^ and the observation that JMJD5 does not catalyse the demethylation of *N*^ε^-methyl lysine residues *in vitro* and in cells.^22–25^

The reduced ability of 2OG derivatives to react with KDM4E compared to JMJD5, FIH, and, in particular, AspH, is in accord with the reported inability of isolated human wildtype KDM4A to employ C4-substituted 2OG derivatives as cosubstrates, including 4-methyl-2OG (8),^47^ which may reflect a need for a catalytically productive binding mode of the 2OG derivative. MALDI-TOF MS assays with the Phe185Gly KDM4A variant and C4-substituted 2OG derivatives indicate that the Phe185 side chain, which is located in proximity of the 2OG C4 methylene unit, possibly impairs efficient reaction of the C4-substituted 2OG derivatives with wildtype KDM4A.^47^ Considering the high sequence and structural similarities of KDM4A and KDM4E, it is likely that the analogous interaction of the Phe186 side chain of KDM4E, which corresponds to Phe185 in KDM4A, with the C4 substituent of 2OG derivatives has a similar effect on the reaction of the 2OG derivatives with KDM4E.^48^ The ability of KDM4E to employ 3-methyl-2OG (1), but not 4-methyl-2OG (8), as a cosubstrate indicates that even apparently minor changes in the 2OG derivative substitution pattern can have substantial effects on the reactivity of 2OG derivatives with 2OG oxygenases, in accord with the reported reactivity differences of 1 and 8 with FIH and AspH (Table 3);^37^ note that 1 has not been investigated as a cosubstrate for KDM4A yet.

### Kinetic studies support the observation that JMJD5 efficiently reacts with 2OG derivatives

**Fig. 3:**
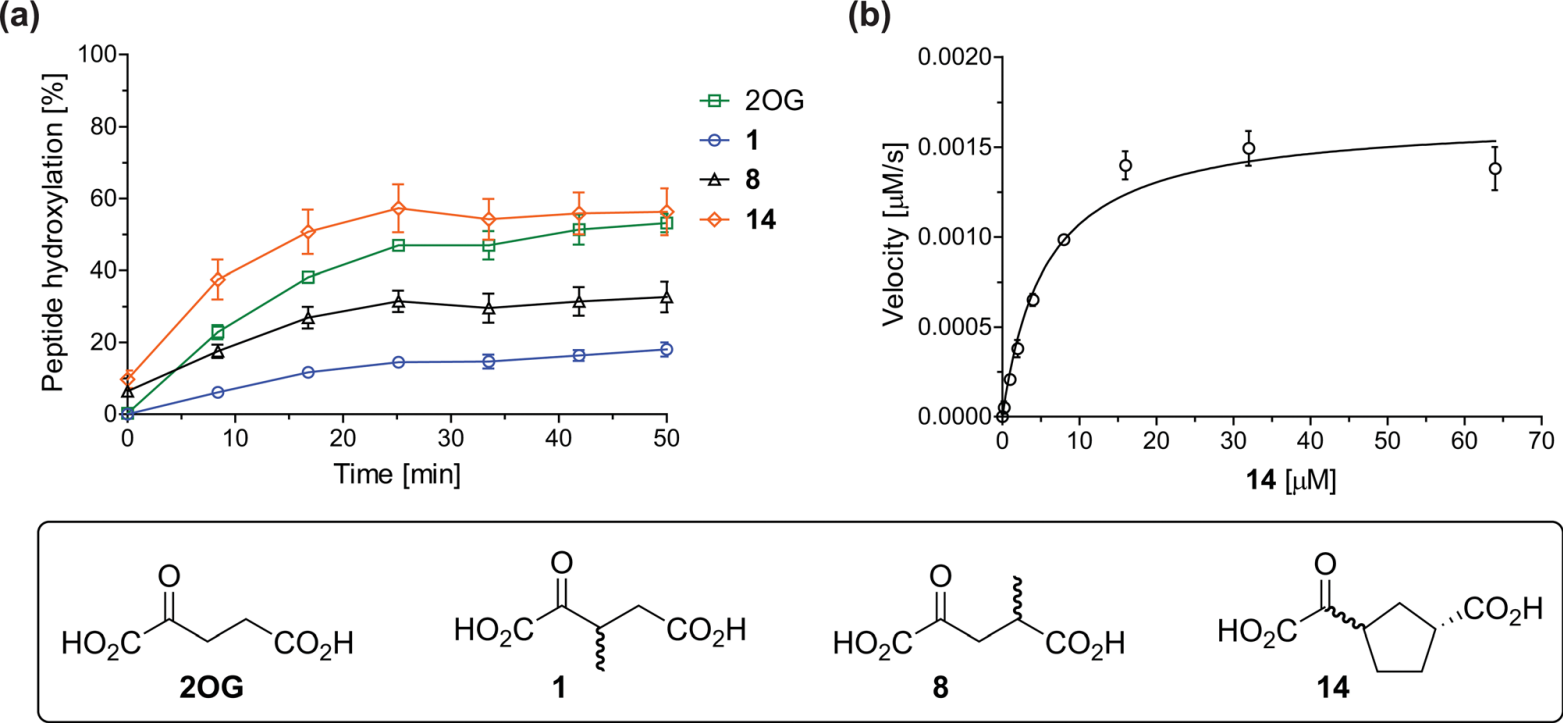
Determination of steady-state kinetic parameters for JMJD5-catalyzed hydroxylations of RPS6_128–148_ using 2OG derivatives as cosubstrates. (a) Time-course for the JMJD5-catalyzed hydroxylation of RPS6_128–148_ using either 2OG (green boxes), 3-methyl-2OG (1, blue circles), 4-methyl-2OG (8, black triangles) or (1*R*)-3-(carboxycarbonyl)cyclopentane-1-carboxylic acid (14, orange diamonds) as cosubstrate. Conditions: 0.15 μM JMJD5, 100 μM LAA, 10 μM Fe(ii), 2.0 μM RPS6_128–148_, and 10 μM of 2OG/2OG derivative in buffer (50 mM MOPS, pH 7.5, 20 °C); (b) determination of the JMJD5 *v*^app^_max_ and *K*^app^_m_ parameters for 14. SPE-MS assays were performed as described in the Experimental section; initial hydroxylation rates used to determine kinetic parameters are shown in Fig. S2 (ESI†). Results are means of three independent runs (*n* = 3; mean ± SD). Measurement times were normalized to the first sample injection analyzed after the addition of JMJD5 to the substrate mixture (*t* = 0 min), by which time low levels of hydroxylation were manifest. 2OG derivatives were prepared from cyanosulfur ylids as racemic mixtures as reported;^44^14 was used as a mixture of diastereomers, dr (*cis* : *trans*) = 1 : 1.^44^

The effect of the 2OG derivatives 1, 8, and 14 (10 μM each) on JMJD5 catalysis was investigated as a function of time using SPE-MS assays with 2OG as a positive control (Fig. 3a). The results reveal that the time course profile of JMJD5-catalyzed RPS6_128–148_ hydroxylation when using 14 as a cosubstrate in the absence of 2OG resembles that of 2OG (10 μM). In both cases, JMJD5-catalyzed RPS6_128–148_ hydroxylation reached ∼50% after 50 min (Fig. 3a), an observation which is remarkable considering that the cyclic structure of 2OG derivative 14 is conformationally rigid compared to the more conformationally mobile 2OG. By contrast, levels of RPS6_128–148_ hydroxylation were substantially lower when using 4-methyl-2OG (8; ∼30% conversion) or 3-methyl-2OG (1; ∼20% conversion) as JMJD5 cosubstrates, in accord with the initial end-point turnover assays (Table S1, ESI†). Nonetheless, the observation that 2OG derivatives 1 and 8 can sustain JMJD5 activity in the absence of 2OG is of interest considering that both compounds are natural products which are present in human nutrition.^49–52^

**Fig. 4:**
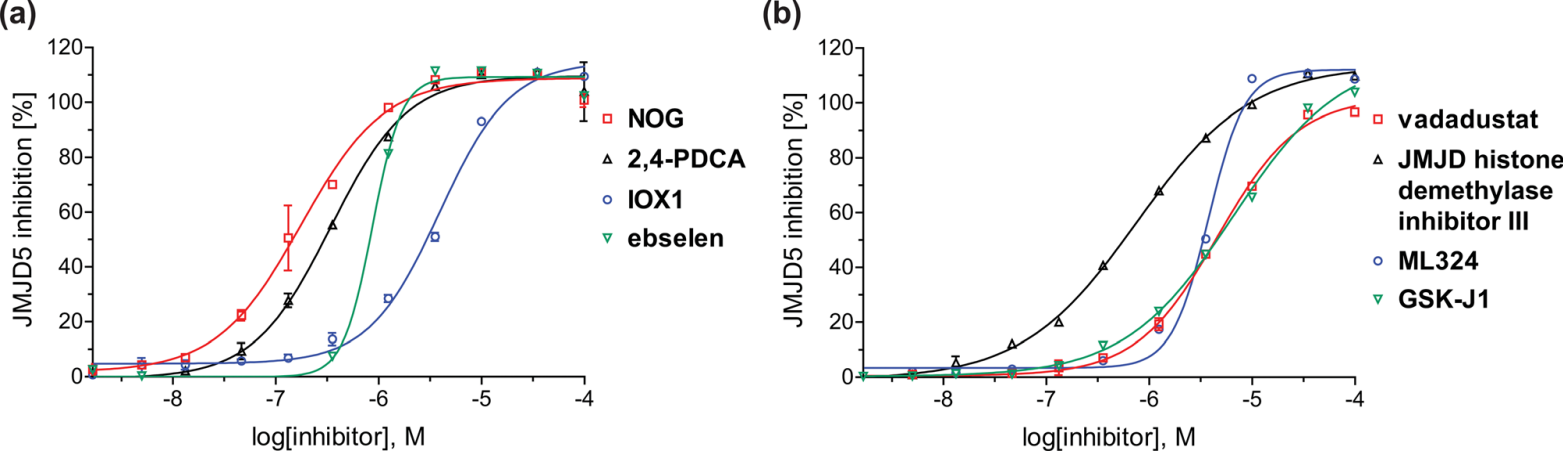
Dose-response curves of efficient JMJD5 inhibitors. Representative dose-response curves used to determine IC_50_-values for (a) NOG (red boxes), 2,4-PDCA (black triangles), IOX1 (blue circles), ebselen (green inverse triangles), and (b) vadadustat (red boxes), JMJD histone demethylase inhibitor III (black triangles), ML324 (blue circles), GSK-J1 (green inverse triangles). Two dose-response curves each composed of technical duplicates were independently determined using SPE-MS JMJD5 inhibition assays (for assay details see Experimental section). Hill coefficients^74^ of the inhibition curves range ∼1, as predicted for single molecules competing with the 2OG for binding JMJD5, with exception of ebselen and ML324 for which they are ≥2.

Kinetic studies were performed to quantify the ability of the 2OG derivative 14 containing a cyclic carbon backbone to act as a JMJD5 cosubstrate and to enable an accurate comparison with 2OG (Table 4). The results reveal that the *k*^app^_cat_ value of JMJD5 for 14 is about twofold higher than that for 2OG, whereas its *K*^app^_m_ value for 14 is ∼19-fold higher than that for 2OG (Table 4, entries i and ii). Assuming that the *K*^app^_m_ values reflect the affinity of JMJD5 for a cosubstrate, the results indicate that JMJD5 binds its natural cosubstrate 2OG with higher affinity than the synthetic 2OG derivative 14. It should be noted, however, that 14 was employed as a diastereomeric mixture composed of the (1*R*,3*S*)- and the (1*R*,3*R*)-diastereomers, owing to the facile epimerization of its stereocenter α to the ketone during synthesis. It may be that JMJD5 preferentially binds one of the two tested diastereomers of 14, potentially resulting in a lower *K*^app^_m_ value for the favored diastereomer; however, docking studies performed with JMJD5 indicate that both the (1*R*,3*S*)- and the (1*R*,3*R*)-diastereomers of 14 can bind to the JMJD5 active site (Fig. S4, ESI†). This proposal is precedented by work with FIH and 14, as a FIH:14:substrate complex crystal structure revealed that the (1*R*,3*S*)-diastereomer of 14, rather than the (1*R*,3*R*)-diastereomer, preferentially binds to FIH.^37^ The divergent trend observed for the *k*^app^_cat_ and *K*^app^_m_ values of JMJD5 for 2OG and 14 results in an approximate tenfold difference in the JMJD5 *k*_cat_/*K*_m_ values of 2OG and 14; thus, the *k*_cat_/*K*_m_ values indicate that 2OG is a ∼10-fold more efficient JMJD5 cosubstrate than 14 (Table 4, entries i and ii).

**Table 4:** SPE-MS steady-state kinetic parameters of JMJD5, FIH, and KDM4E for 2OG and 2OG derivative 14^a^

	Cosubstrate	2OG oxygenase	*k* ^app^_cat_ [s^−1^]	*K* ^app^_m_ [μM]	*k*_cat_/*K*_m_ [mM^−1^ s^−1^]
i	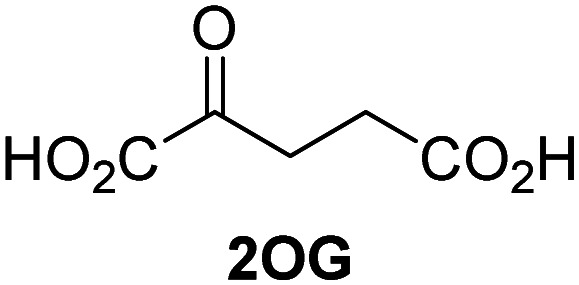	JMJD5^b^	5.6 × 10^−3^ ± 0.2 × 10^−3^	0.29 ± 0.04	19.3 ± 5.8
ii	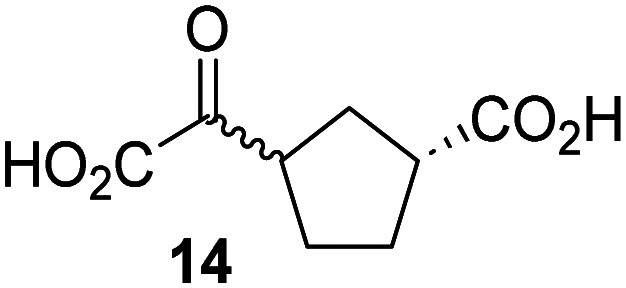	JMJD5^b^	11.3 × 10^−3^ ± 0.7 × 10^−3^	5.6 ± 0.7	2.1 ± 0.3
iii	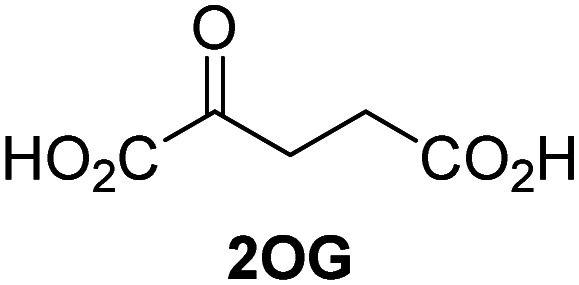	FIH^37^ c	0.04 ± 0.01	0.8 ± 0.1	47.6 ± 12.5
iv	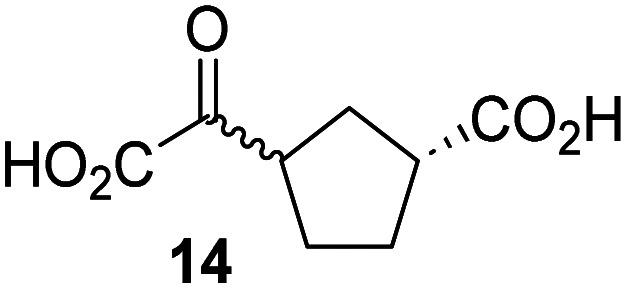	FIH^37^ c	0.01 ± 0.001	14.8 ± 2.6	0.7 ± 0.2
v	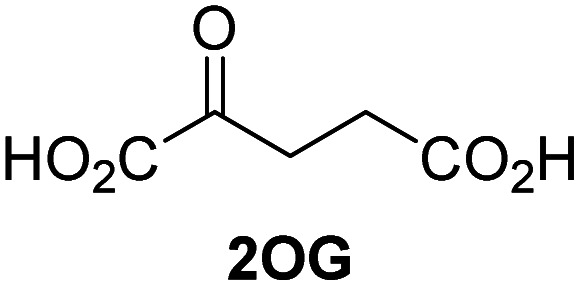	KDM4E^d^	0.14 ± 0.01	4.2 ± 1.1	33.4 ± 9.1
vi	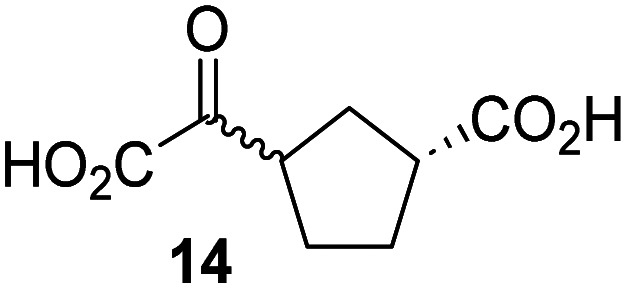	KDM4E^d^	No reaction	No reaction	No reaction

a The results are means of three independent runs (*n* = 3; mean ± SD).

b Determined using JMJD5 (0.15 μM) and RPS6_128–148_ as substrate (4.0 μM), as described in the Experimental section.

c Reported values were obtained using SPE-MS assays which employed FIH (0.15 μM) and HIF-1α_788–822_^42^ as a substrate (5.0 μM).^37^

d Determined using 0.15 μM KDM4E and ARTAQTARK(me3)STGGIA (a histone 3 K9(me3) derivative)^41^ as substrate (10 μM) (Fig. S3).

As previously reported, FIH can also employ the carbocyclic 2OG derivative 14 as a cosubstrate, however, 14 is a substantially less efficient FIH cosubstrate than 2OG as reflected by an ∼70-fold difference in the FIH *k*_cat_/*K*_m_ values (Table 4, entries iii and iv).^37^ Thus, the comparison of the JMJD5, FIH, and KDM4E *k*_cat_/*K*_m_ values of 2OG and 14 reveals that 2OG is a more efficient cosubstrate for FIH and KDM4E than for JMJD5, whereas 14 is a more efficient cosubstrate for JMJD5 than for FIH and KDM4E (Table 4 and Fig. S3, ESI†). This observation highlights the potential of 14 (and other 2OG derivatives) to selectively alter the relative reaction rates of 2OG oxygenases.

### JMJD5 inhibition studies

A high-throughput JMJD5 SPE-MS inhibition assay was developed to help enable the identification of small-molecule JMJD5 inhibitors for functional assignment studies. The SPE-MS inhibition assay was employed to determine half-maximum inhibitory concentrations (IC_50_ values) of reported 2OG oxygenase inhibitors for JMJD5. Initially, a set of broad-spectrum 2OG oxygenase inhibitors was investigated for JMJD5 inhibition, including *N*-oxalylglycine (NOG),^53^ pyridine-2,4-dicarboxylic acid (2,4-PDCA),^53^ IOX1,^54^ and ebselen^53^ (Table 5, entries i–iv). Efficient JMJD5 inhibition was observed for NOG and 2,4-PDCA (IC_50_ ∼ 0.2 and ∼0.3 μM, respectively; Table 5), while IOX1 was about an order of magnitude less potent. Ebselen also inhibits JMJD5 efficiently (IC_50_ ∼ 0.7 μM; Table 5, entry iv), likely by covalent reaction with one or multiple of the eleven cysteine residues in JMJD5, as observed for other cysteine residue-containing proteins including 2OG oxygenases.^55–59^

**Table 5:** Inhibition of JMJD5 by reported small-molecule 2OG oxygenase inhibitors^a^

	2OG oxygenase inhibitor	IC_50_ [μM]		2OG oxygenase inhibitor	IC_50_ [μM]
i	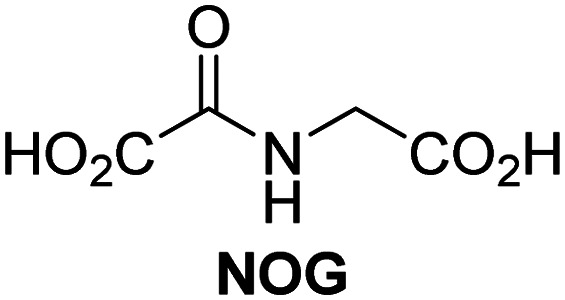	0.15 ± 0.02	ix	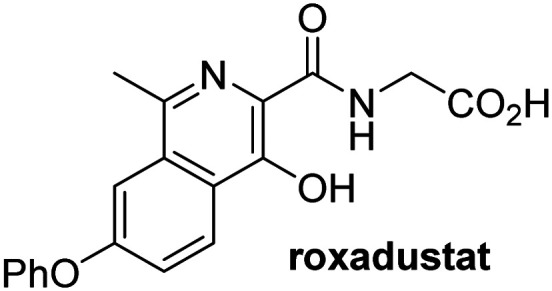	46.3 ± 3.0
ii	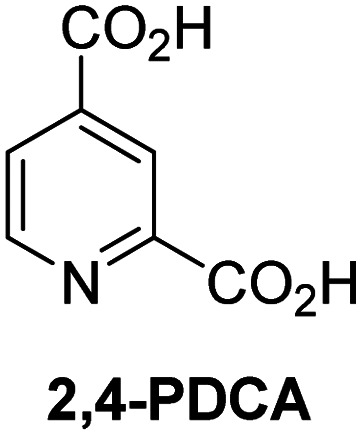	0.33 ± 0.07	x	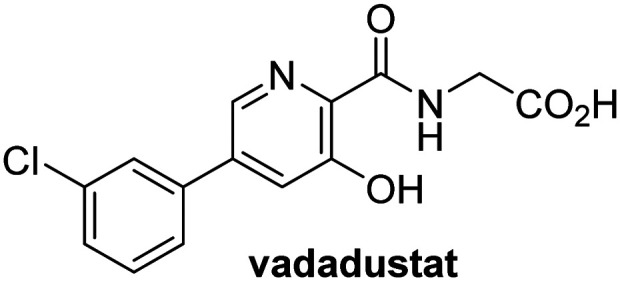	4.1 ± 0.4
iii	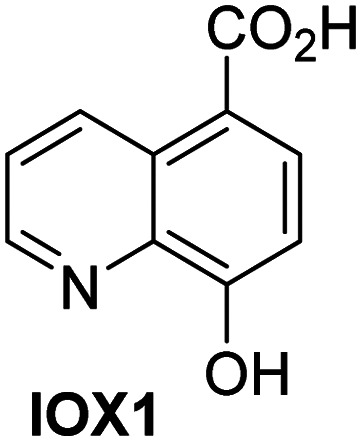	2.6 ± 0.1	xi	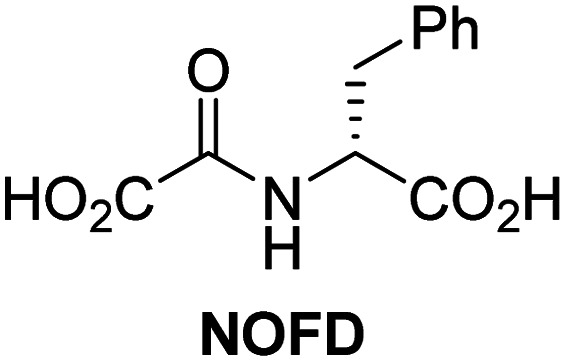	>100
iv	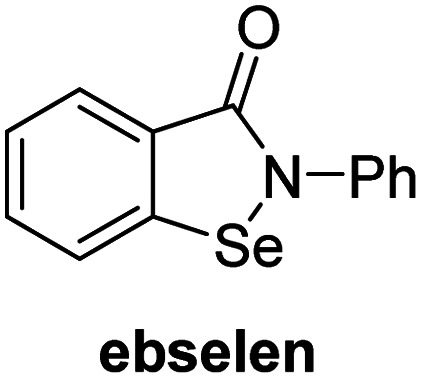	0.69 ± 0.15	xii	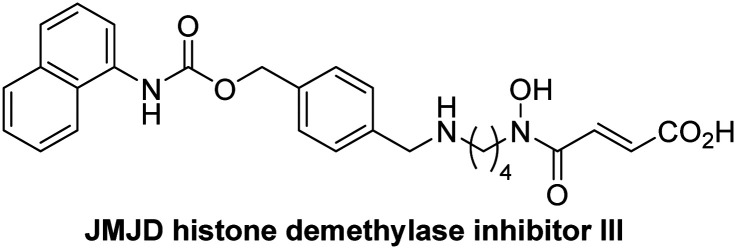	0.6 ± 0.1
v	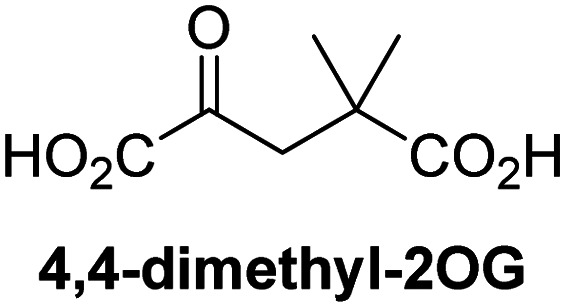	>100	xiii	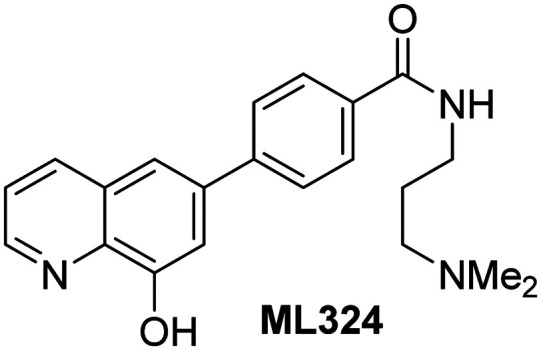	3.0 ± 0.5
vi	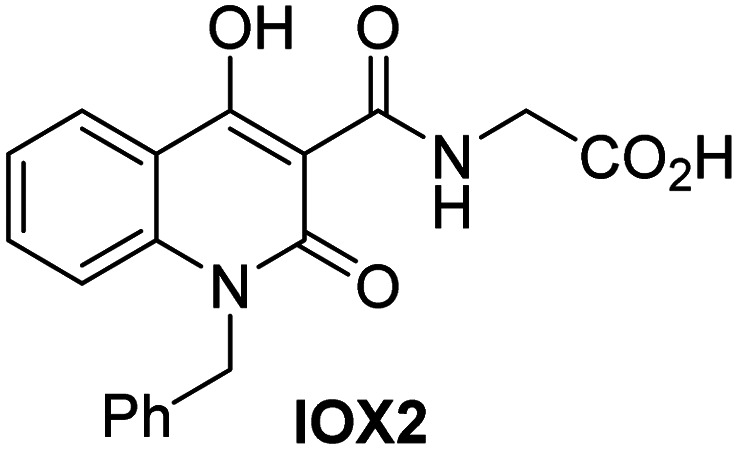	17.8 ± 5.6	xiv	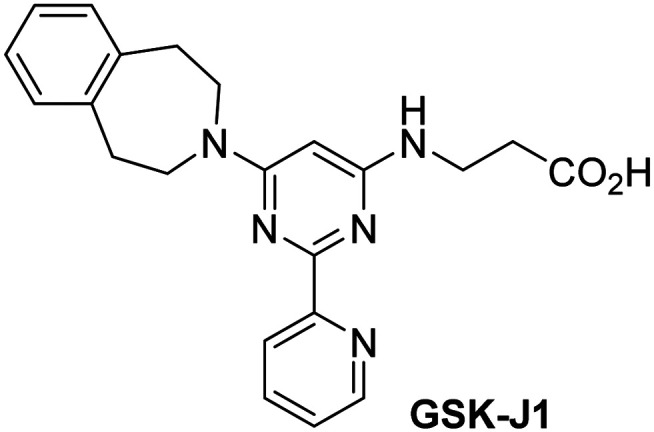	4.4 ± 0.5
vii	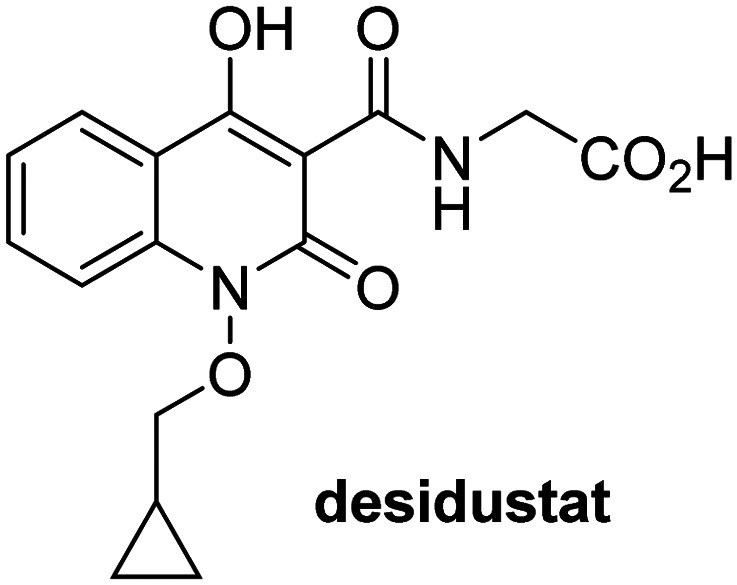	37.2 ± 6.0	xv	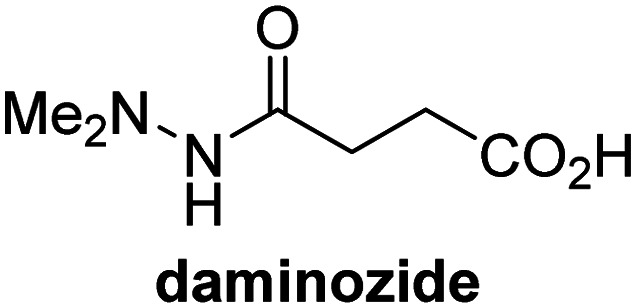	>100
viii	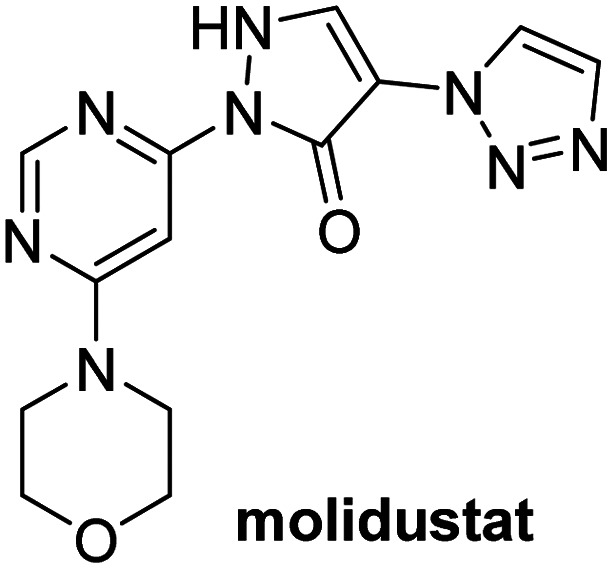	29.5 ± 1.6	xvi	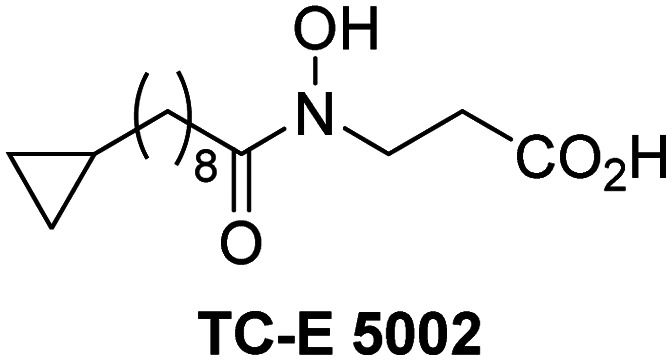	14.4 ± 0.1

a SPE-MS inhibition assays were performed as described in the Experimental section employing JMJD5 (0.15 μM), 2OG (2.0 μM), Fe(ii) (2.0 μM), LAA (100 μM), and RPS6_128–148_ (2.0 μM) in buffer (50 mM MOPS, pH 7.5, 20 °C). The results are means of two independent runs, each composed of technical duplicates (*n* = 2; mean ± SD). *Z*′-Factors of the assays were >0.8 indicating excellent assay robustness.^61^ Representative dose-response curves of efficient JMJD5 inhibitors are shown in Fig. 4.

Reported crystal structures of JMJD5 in complex with NOG or 2,4-PDCA reveal that these broad-spectrum 2OG oxygenase inhibitors modulate JMJD5 catalysis by competition with 2OG for binding to the active site.^35^ 2OG derivatives/competitors may inhibit JMJD5 in a similar manner, as precedented by their reported inhibition of FIH and AspH.^37,44^ Thus, a set of 34 synthetic 2OG derivatives/competitors was investigated for JMJD5 inhibition; however, by contrast with the reported results for AspH and FIH,^37,44^ no efficient inhibitor (*i.e.* IC_50_ < 20 μM) of JMJD5 was identified (Table S2, ESI†). The inhibition results are in accord with the reduced ability of the 2OG derivatives to sustain JMJD5 catalysis as cosubstrates than AspH catalysis (Table 3), which reflects the crystallographic observations that the JMJD5 2OG binding pocket is more compact than that of FIH and AspH.^20,32^ Nonetheless, the results highlight the potential of 2OG competitors for selective 2OG oxygenase inhibition, *e.g.* 4,4-dimethyl-2OG (9) inhibits AspH, but not JMJD5 or FIH (Table S2, ESI†).^37,44^ It has also been reported that 4,4-dimethyl-2OG (9) does not inhibit human cancer-associated variants of isocitrate dehydrogenase (IDH);^60^ these IDH variants employ 2OG as a substrate (but not 9^60^), however, they are functionally and structurally unrelated to human 2OG oxygenases.

Interestingly, while none of the tested 34 2OG derivatives/competitors were able to sustain KDM4E catalysis as cosubstrates alternative to 2OG, three of them were potent KDM4E inhibitors (*i.e.* IC_50_ < 20 μM), *i.e.* 3-(9,9-dimethyl-9*H*-fluoren-2-yl)methyl-2OG (7, IC_50_ ∼ 6.1 μM), 4-methyl-2OG (8, IC_50_ ∼ 6.6 μM), and 4-(2-naphthyl)methyl-2OG (23, IC_50_ ∼ 6.9 μM), indicating that both C3 and C4 substituted 2OG derivatives can bind to the KDM4E active site (Table S2, ESI†). Note that 2OG derivative 7, which bears a bulky fluorenyl derivative at the C3 position, has also been found to efficiently inhibit both FIH and AspH (Table S2, ESI†).^37,44^ The observation that the C4 substituted 2OG derivatives 8 and 23 inhibit KDM4E is in accord with previous results showing that structurally related NOG derivatives, bearing substituents on the glycine methylene unit (corresponding to the 2OG C4 position), efficiently inhibit KDM4A and KDM4E.^45,46^ Thus, the lack of cosubstrate activity of the tested 34 2OG derivatives with KDM4E does not reflect the observation that 2OG derivatives can bind to the KDM4E active site, but indicates that other factors, such as *e.g.* the conformational flexibility of the 2OG derivative when bound to the active site and/or disruption of the catalytic cycle, determine productive turnover.

In addition to broad-spectrum 2OG oxygenase inhibitors, small-molecules which display higher levels of selectivity for specific 2OG oxygenases or subclasses of 2OG oxygenases were investigated for JMJD5 inhibition. Firstly, the HIF-α prolyl residue hydroxylase (PHD) inhibitors IOX2,^62^ roxadustat,^63^ molidustat,^64^ vadadustat,^65^ and desidustat,^66^ some of which are approved for clinical use to treat chronic kidney disease-associated anemia,^67^ were tested (Table 5, entries vi–x). Consistent with the MALDI-TOF MS-based results,^35^ in general, these PHD inhibitors did not inhibit JMJD5 efficiently in the SPE-MS assay (IC_50_ > 15 μM; Table 5, entries vi–ix), with the notable exception of vadadustat (IC_50_ ∼ 4.1 μM; Table 5, entry x). Vadadustat was identified as a weak JMJD5 inhibitor using MALDI-TOF MS assays;^35^ however, the obtained IC_50_-value was higher (∼54 μM), reflecting the higher sensitivity of SPE-MS assays for inhibition studies. In general, the selectivity of vadadustat for inhibition of the PHDs over other 2OG oxygenases typically appears to be lower compared to other PHD inhibitors, *e.g.*, vadadustat also inhibits AspH with similar potency as JMJD5.^56^*N*-Oxalyl-d-phenylalanine (NOFD), which is a reported selective FIH inhibitor,^68^ does not inhibit JMJD5 in the concentration range tested (Table 5, entry xi).

The reported JmjC KDM4 subfamily-specific inhibitors JMJD histone demethylase inhibitor III (the acid form of the methyl ester prodrug methylstat)^69^ and ML324^70^ efficiently inhibited JMJD5 (Table 5, entries xii and xiii), in accord with the reported inhibition of JMJD5 by ML324 observed using MALDI-TOF MS assays.^35^ JMJD histone demethylase inhibitor III inhibits JMJD5 with similar potency as the broad-spectrum 2OG oxygenase inhibitor ebselen (IC_50_ ∼ 0.6 μM; Table 5, entry xii). Although JMJD histone demethylase inhibitor III is reported to be a specific inhibitor of the KDM4 subfamily, it apparently inhibits JMJD5 more efficient than the KDM4 oxygenases; note, however, that its reported IC_50_-values for KDM4 oxygenases were obtained using assays other than SPE-MS.^69^ GSK-J1, a reported inhibitor of the KDM6 subfamily,^71^ inhibits JMJD5, albeit not as potently as the broad-spectrum 2OG oxygenase inhibitors (IC_50_ ∼ 4.4 μM; Table 5, entry xiv). Unlike the investigated KDM4 and KDM6 inhibitors, the reported KDM2/7 inhibitors daminozide^72^ and TC-E 5002^73^ do not inhibit JMJD5 efficiently (IC_50_ >100 and ∼14.4 μM, respectively; Table 5, entries xv and xvi).

## Conclusions

Efficient assays which monitor the activity of isolated recombinant human JMJD5 *in vitro* are needed to complement cellular and biological functional assignment studies considering the contradictory reports on the biochemical role(s) of JMJD5.^19–25,32^ A JMJD5 SPE-MS assay was developed and applied to determine the kinetic parameters for JMJD5; the SPE-MS assay results also confirm previous studies using MALDI-TOF MS assays that JMJD5 is an arginine C3 hydroxylase.^32,35^ In general, the JMJD5 kinetic parameters are in the range of those reported for other human 2OG oxygenases (Table 2), however, the *k*^app^_cat_ values appear to be ∼10- to ∼100-fold lower than those reported for other human 2OG oxygenases suggesting that RPS6-derived oligopeptides may not be ideal substrates for isolated JMJD5 compared to full-length folded RPS6 and/or that JMJD5 catalyses the arginyl-residue hydroxylation of other, yet unidentified, substrates more efficiently. The proposal that JMJD5 may accept substrates other than RCCD1 or RPS6 is precedented by the substrate promiscuity of some, but apparently not all, human 2OG oxygenases, including FIH, AspH, and JMJD6.^75–79^

The JMJD5 SPE-MS assays were employed to investigate the effect of 2OG derivatives on JMJD5 catalysis (Tables S1 and S2, ESI†); six 2OG derivatives were identified that sustain JMJD5 activity in the absence of 2OG (Fig. 3a), amongst them 3- and 4-methyl 2OG (1 and 8) which both are natural products present in human nutrition.^49–52^ Our results thus support the proposal that 2-oxoacids other than 2OG could, at least in principle, be preferred cosubstrates of certain 2OG oxygenases *in vivo* considering that 1 and 8 have been shown to sustain the catalytic activity of other isolated human 2OG oxygenases with different selectivity profiles.^37,44^ In future work, it would be of interest to explore whether 2OG derivatives can alter the substrate selectivity of 2OG oxygenases that accept different substrates, *e.g.* the KDM4 JmjC demethylases, which accept different histone-based *N*-methylation states of Lys and Arg residues.^80^

The observation that a 2OG derivative with a conformationally constrained cyclic carbon backbone, *i.e.* (1*R*)-3-(carboxycarbonyl)cyclopentane-1-carboxylic acid (14), can efficiently replace 2OG in JMJD5 catalysis (and, to a lesser extent, in FIH and AspH catalysis; Table 3, entry xv) is particularly notable, especially given many other C3 and/or C4 substituted 2OG derivatives are not substrates. It appears feasible that conformationally constrained derivatives of 2OG may be identified which could be more efficient JMJD5 cosubstrates than 2OG itself.

The inhibition studies show that some 2OG derivatives are (selective) inhibitors of 2OG oxygenases, including for AspH, FIH, and KDM4E (Table S1, ESI†). Although we have not defined the modes of action of the inhibitors, the results imply scope for mechanism-based inhibition rather than simple active site blockade.

The geometry of 2OG oxygenase active sites can differ substantially as shown by crystallographic analyses^81^ which, however, may not necessarily reflect the solution geometries with complete accuracy, as implied by modelling studies.^82–84^ 2OG derivatives thus appear to be attractive tools to investigate the active site requirements of 2OG oxygenases in solution, which may complement crystallographic studies. The SPE-MS assays reveal that JMJD5 reacts with a similar efficiency with certain 2OG derivatives as does FIH and with a greater efficiency than KDM4E, but less efficiently than AspH which can also use a structurally more diverse set of 2OG derivatives as cosubstrates, including those derivatives with an aromatic scaffold (Table 3 and Table S1, ESI†).^37,44^ The reduced reactivity of JMJD5 with 2OG derivatives may reflect the crystallographically-observed relatively compact 2OG binding site of JMJD5, at least as compared to some other 2OG oxygenases such as AspH.^32^ The hypoxia sensing PHDs also have compact 2OG binding sites resulting in formation of a stable PHD:Fe(ii):2OG complex which manifests in low levels of uncoupled 2OG turnover,^85,86^ as also reported for JMJD5.^32^

The observation that neither 3- nor 4-methyl-2OG (1 and 8) sustain JMJD5-catalysed RPS6_128–148_ hydroxylation as efficiently as 2OG (Fig. 3a) is precedented by the reduced reactivity of 1 and 8 with FIH compared to 2OG (Table 3),^37^ supporting the crystallographic evidence that JMJD5 and FIH have similar overall active site geometries.^24,25,32^ By contrast, based on *k*_cat_/*K*_m_ values, 3-methyl-2OG (1) is reported to be a more efficient cosubstrate for isolated recombinant AspH than 2OG.^44^ 2OG derivatives 8, 10, 11, and 14 are cosubstrates of both JMJD5 and FIH, but not of KMD4E, supporting the assignment of JMJD5 as a hydroxylase rather than a lysine demethylase. The combined results show that the reactivity profiles of 2OG oxygenases with 2OG derivatives can differ substantially, likely because of the different geometries of their 2OG binding pockets,^81^ which may indirectly inform on 2OG oxygenase function.

SPE-MS assays have been of utility for profiling and developing 2OG oxygenase inhibitors, as reported results with KDM4s and other KDM subfamilies,^87–90^ PHD2,^91^ FIH,^37^ ribosomal oxygenase 2 (RIOX2),^92^ and AspH^93,94^ manifest. The described JMJD5 SPE-MS assays should facilitate research efforts directed at identifying selective small-molecule JMJD5 inhibitors which may be of value to dissect the roles of JMJD5 in healthy and cancer biology. So far, mostly broad-spectrum 2OG oxygenase inhibitors have been identified to inhibit JMJD5, *i.e.* NOG, 2,4-PDCA, and ebselen (Table 5), which may be *per se* of limited utility for cellular functional assignment studies because of their lack of selectivity. However, NOG is commonly used in cellular studies to induce a cellular state mimicking hypoxia, likely *via* PHD2 inhibition, which triggers HIF upregulation.^53^ Nonetheless, the broad-spectrum inhibitors may be valuable lead structures for the design of more selective JMJD5 inhibitors, as precedented by an NOG derivative which is a selective FIH inhibitor, *i.e.* NOFD,^68^ and by 2,4-PDCA derivatives which display improved selectivity profiles for AspH inhibition.^95^ In this regard, it is notable that JMJD5 contains an *N*-methylarginine binding pocket that could be exploited for the development of selective inhibitors.^20^

Apart from their potential to enable the identification and design of efficient JMJD5 inhibitors, the SPE-MS inhibition assays are of use to investigate the effects of reported selective inhibitors of other 2OG oxygenase on JMJD5 catalysis, which is important to determine potential off-target effects and thus to develop safer therapeutics for use in humans. For instance, the JMJD5 inhibition assays reveal that the PHD inhibitor roxadustat, which is the active pharmaceutical ingredient of a human chemotherapeutic used to treat chronic kidney disease-associated anemia,^63,67^ does not inhibit JMJD5 at substantial levels (Table 5, entry ix). By contrast, the reported KDM4 subfamily-specific inhibitor JMJD histone demethylase inhibitor III^69^ also efficiently inhibits JMJD5 (Table 5, entry xii), further demonstrating that care needs to be taken when interpreting cellular studies performed with 2OG oxygenase inhibitors.

## Experimental

### General information

Inhibitors were commercially-sourced and used as received, 2OG derivatives were synthesized as reported.^37,44^ For JMJD5 assays, cosubstrate/cofactor stock solutions (l-ascorbic acid, LAA: 50 mM in Milli Q (MQ)-grade water; 2-oxoglutarate, 2OG: 10 mM in MQ-grade water; ammonium iron(ii) sulfate hexahydrate, FAS, (NH_4_)_2_Fe(SO_4_)_2_·6H_2_O: 400 mM in 20 mM HCl diluted to 1 mM in MQ-grade water) were freshly prepared from commercially-sourced solids (Sigma Aldrich) on the day the SPE-MS assays were performed.

### Production and purification of human JMJD5

The sequence coding for full-length JMJD5 (M1–S416) was sub-cloned into the bacterial expression vector pNH-Trxt, which encodes for an N-terminal His_6_-thioredoxin-TEV site tag. Plasmids were transformed into *Escherichia coli* BL21(DE3) cells; transformed cells were grown overnight at 37 °C in Terrific Broth media (12 × 10 mL) containing 100 μg mL^−1^ kanamycin. The overnight cell culture was used to inoculate 12 L of Terrific Broth media containing 100 μg mL^−1^ kanamycin; cells were grown at 37 °C until an OD_600_ of ∼1.0. Then, the temperature was reduced to 18 °C, and protein production was induced by the addition of 0.5 mM IPTG; cells were shaken overnight. Cells were centrifuged, re-suspended in lysis buffer (50 mM Tris, pH 8, 500 mM NaCl, 20 mM imidazole, 0.5 mM tris(2-carboxyethyl)phosphine (TCEP), 5%_v/v_ glycerol), which contained a 1 : 2000 EDTA-free protease inhibitor cocktail (Roche Diagnostics Ltd), and lysed at 4 °C using a high-pressure cell breaker (EmulsiFlex-C5, Avestin; three passages). The lysate was centrifuged and loaded onto a Ni(ii) NTA affinity chromatography column. After extensive washing with lysis buffer, His_6_-thioredoxin-tagged JMJD5 was eluted in lysis buffer containing imidazole (300 mM). His_6_-thioredoxin-tagged JMJD5 was purified further using size-exclusion chromatography (S200 gel filtration column attached to an ÄKTA Xpress system) and elution buffer (50 mM Tris, pH 8, 150 mM NaCl, 0.5 mM TCEP, 5%_v/v_ glycerol). His_6_-thioredoxin-tagged JMJD5 was >95% pure as analyzed by SDS-PAGE and SPE-MS analysis; fresh aliquots of His_6_-thioredoxin-tagged JMJD5 were used for all assays.

### JMJD5 substrates

JMJD5 substrates were based on the sequence of the reported substrate proteins,^32^*i.e.* RCCD1 (RCCD1 amino acids 134–150, RCCD1_134–150_: LPLLPCARAYVSPRAPF; JMJD5 catalyses the C3 hydroxylation of R141^32^) and RPS6 (RPS6 amino acids 128–148, RPS6_128–148_: TVPRRLGPKRASRIRKLFNLS; JMJD5 catalyses the hydroxylation of R137^32^). Peptides were synthesized by solid-phase peptide synthesis and purified by GL Biochem (Shanghai) Ltd (Shanghai, China); all peptides were prepared with C-terminal amides.

### JMJD5 SPE-MS assays

JMJD5 turnover assays for time course and kinetic experiments were performed in 96-well polypropylene assay plates (Greiner) with either 1.0 or 0.5 mL total reaction volume using the concentrations given in the manuscript or ESI,† and JMJD5-catalysed substrate hydroxylation was directly monitored using SPE-MS. MS-analyses were performed using a RapidFire RF 365 high-throughput sampling robot (Agilent) attached to an iFunnel Agilent 6550 accurate mass quadrupole time-of-flight (Q-TOF) mass spectrometer operated in the positive ionization mode. The RapidFire RF 365 high-throughput sampling robot was programmed to aspirate samples from the reaction mixture at the indicated time intervals. Assay samples were aspirated under vacuum for 0.6 s and loaded onto a C4 solid phase extraction (SPE) cartridge. After loading, the C4 SPE cartridge was washed with 0.1%_v/v_ aqueous formic acid to remove non-volatile buffer salts (5.5 s, 1.5 mL min^−1^). The peptide was eluted from the SPE cartridge with 0.1%_v/v_ aqueous formic acid in 80/20_v/v_ acetonitrile/water into the mass spectrometer (5.5 s, 1.5 mL min^−1^) and the SPE cartridge re-equilibrated with 0.1%_v/v_ aqueous formic acid (0.5 s, 1.5 mL min^−1^). The mass spectrometer was operated using the MassHunter Workstation B.08.00 software (Agilent), the mass spectrometer parameters were: capillary voltage (4000 V), nozzle voltage (1000 V), fragmentor voltage (365 V), gas temperature (280 °C), gas flow (13 L min^−1^), sheath gas temperature (350 °C), sheath gas flow (12 L min^−1^). The *m*/*z* +5 (for RPS6_128–148_) or +3 (for RCCD1_134–150_) charge states of the peptide (substrate) and the hydroxylated peptide (product) were used to extract ion chromatogram data, peak areas were integrated using RapidFire Integrator 4.3.0 (Agilent). Data were exported into Microsoft Excel and used to calculate the % conversion of the hydroxylation reaction using the equation: % conversion = 100 × (integral product peptide)/(integral substrate peptide + integral product peptide).

### SPE-MS JMJD5 inhibition assays

Solutions of the small-molecules (100% DMSO) were dry dispensed across 384 well polypropylene V-bottom assay microplates (Greiner) in an approximately three-fold and 11-point dilution series (100 μM inhibitor top concentration; the final DMSO assay concentration was kept constant at 0.5%_v/v_) using an ECHO 550 acoustic dispenser (Labcyte). DMSO and 2,4-PDCA were used as negative and positive inhibition controls, respectively. Each reaction was performed in technical duplicates in adjacent wells of the assay plates; additionally, assays were performed in two independent duplicates on different days using different inhibitor solutions.

The Enzyme Mixture (25 μL per well), containing 0.3 μM His_6_-thioredoxin-tagged JMJD5 in buffer (50 mM MOPS, pH 7.5), was dispensed across the inhibitor-containing 384-well plates with a multidrop dispenser (ThermoFischer Scientific) at 20 °C under an ambient atmosphere. The plates were subsequently centrifuged (1000 rpm, 5 s) and incubated for 15 min at 20 °C. The Substrate Mixture (25 μL per well), containing RPS6_128–148_ (4.0 μM), LAA (200 μM), 2OG (4.0 μM), and FAS (4.0 μM) in buffer (50 mM MOPS, pH 7.5), was added using the multidrop dispenser. The plates were centrifuged (1000 rpm, 5 s) and, after incubating for 30 min, the enzyme reaction was stopped by the addition of 10%_v/v_ aqueous formic acid (5 μL per well). The plates were then centrifuged (1000 rpm, 30 s) and analyzed by MS.

MS-analyses were performed using a RapidFire RF 365 high-throughput sampling robot (Agilent) attached to an iFunnel Agilent 6550 accurate mass quadrupole time-of-flight (Q-TOF) mass spectrometer as described above in the SPE-MS assay development section. The % conversion of the hydroxylation reaction was calculated as described above; from the raw data, dose-response curves (normalized to 2,4-PDCA and DMSO controls) were obtained by non-linear regression (GraphPad Prism 5), which were used to determine IC_50_-values. The standard deviation (SD) of two independent IC_50_ determinations (*n* = 2) was calculated using GraphPad Prism 5. *Z*′-Factors were calculated according to the cited literature using Microsoft Excel.^61^

### KDM4E SPE-MS assays

KDM4E turnover and inhibition assays were performed as reported using recombinant human KDM4E and a histone 3 K9(me3) derivative (*i.e.* ARTAQTARK(me3)STGGIA)^41^ as substrate.^95^

## Conflicts of interest

There are no conflicts to declare.

## Supplementary Material

CB-004-D2CB00249C-s001
