# Supplementary material for: Kinetic and inhibition studies on human Jumonji-C (JmjC) domain-containing protein 5
Source: RSC Chem Biol. 2023 Mar 20;4(6):399–413. doi: 10.1039/d2cb00249c (PMC10246557; doi:10.1039/d2cb00249c)
Supplement: CB-004-D2CB00249C-s001 [file CB-004-D2CB00249C-s001.pdf]

Electronic Supplementary Material (ESI) for RSC Chemical Biology.
This journal is © The Royal Society of Chemistry 2023

Supplementary Information

Kinetic and inhibition studies on human Jumonji-C (JmjC) domain-containing protein 5

Anthony Tumber[1,#], Eidarus Salah[1,#], Lennart Brewitz[1,#,*], Thomas P. Corner[1], and Christopher J. Schofield[1,*]

[1]Chemistry Research Laboratory, Department of Chemistry and the Ineos Oxford Institute for Antimicrobial Research, University of Oxford, 12 Mansfield Road, OX1 3TA, Oxford, United Kingdom.

#These authors contributed equally to this work.

* christopher.schofield@chem.ox.ac.uk and lennart.brewitz@chem.ox.ac.uk

Table of contents

1. Supplementary figures 2-8
2. Supplementary tables 9-13
3. References 13

1. Supplementary figures

Supplementary Figure S1. Rates of JMJD5-catalyzed RPS6$_{128-148}$ hydroxylations used to determine kinetic parameters (continues on the following two pages). Maximum velocities (v_{max}^{app}) and Michaelis constants (K_m^{app}) of JMJD5 were determined in independent triplicates, monitoring the JMJD5-catalyzed hydroxylation of the RPS6$_{128-148}$ substrate peptide (TVPRRLGPKRASRIRKLFNLS; JMJD5 catalyses the hydroxylation of R137[1]) by SPE-MS, as described (Experimental Section). Conditions: 0.15 µM JMJD5 in buffer (50 mM MOPS, pH 7.5, 20 °C). Measurement times were normalized to the first sample injection analyzed after the addition of JMJD5 to the Substrate Mixture (t = 0 s), by which time low levels of RPS6$_{128-148}$ hydroxylation were manifest. Data are shown as the mean of three independent runs (n = 3; mean ± standard deviation, SD).

(**a**) Time course of the JMJD5-catalyzed hydroxylation reaction of the RPS6$_{128-148}$ peptide for the shown concentrations of 2OG using RPS6$_{128-148}$ (4.0 µM), L-ascorbic acid (100 µM), and ammonium iron(II) sulfate hexahydrate (20 µM, $(NH_4)_2Fe(SO_4)_2 \cdot 6H_2O$); and (**b**) hydroxylation rates used to determine kinetic parameters of JMJD5 for 2OG.

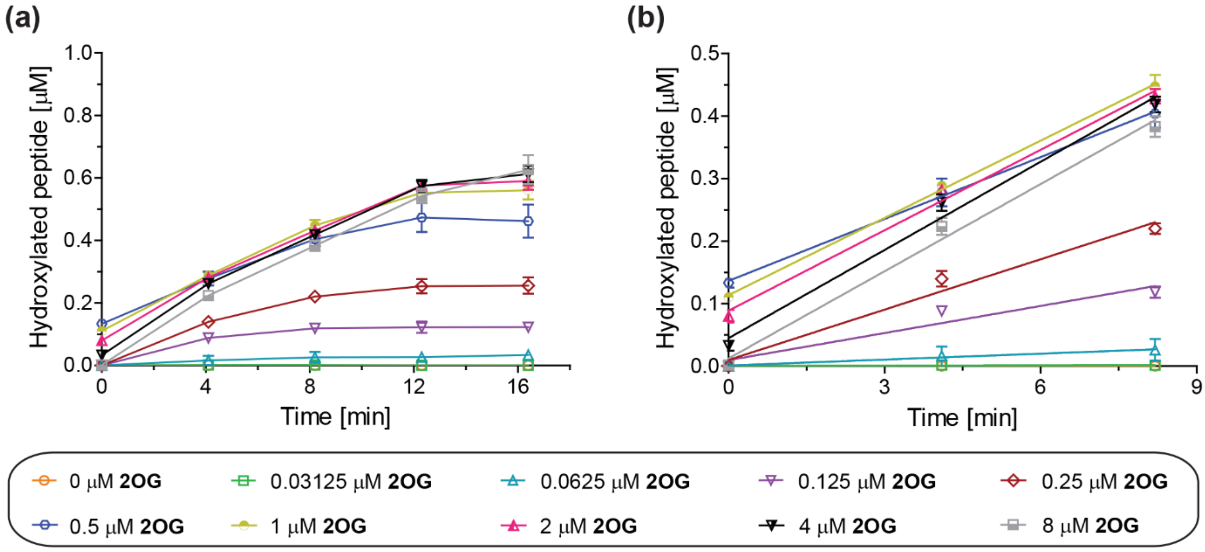

(**c**) Time course of the JMJD5-catalyzed hydroxylation reaction of the $RPS6_{128-148}$ peptide for the shown concentrations of L-ascorbic acid (LAA) using $RPS6_{128-148}$ (4.0 µM), 2OG (20 µM), and ammonium iron(II) sulfate hexahydrate (20 µM, $(NH_4)_2Fe(SO_4)_2{\cdot}6H_2O$); and (**d**) hydroxylation rates used to determine kinetic parameters of JMJD5 for LAA.

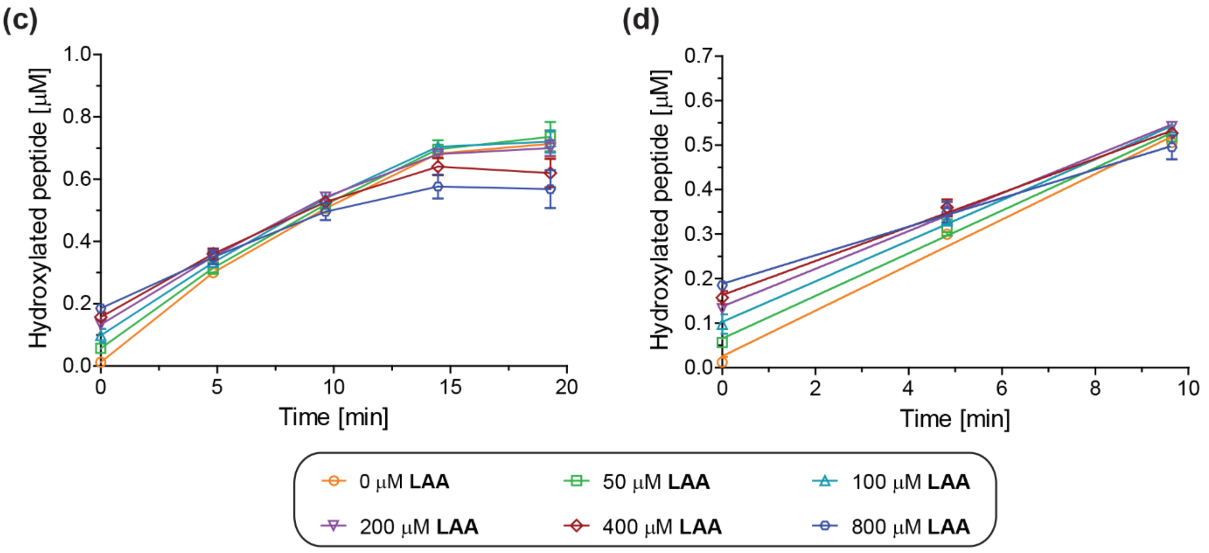

(**e**) Time course of the JMJD5-catalyzed hydroxylation reaction of the $RPS6_{128-148}$ peptide for the shown concentrations of Fe(II) in the presence of L-ascorbic acid using $RPS6_{128-148}$ (4.0 µM), L-ascorbic acid (100 µM), and 2OG (20 µM); and (**f**) hydroxylation rates used to determine kinetic parameters of JMJD5 for Fe(II) in the presence of L-ascorbic acid.

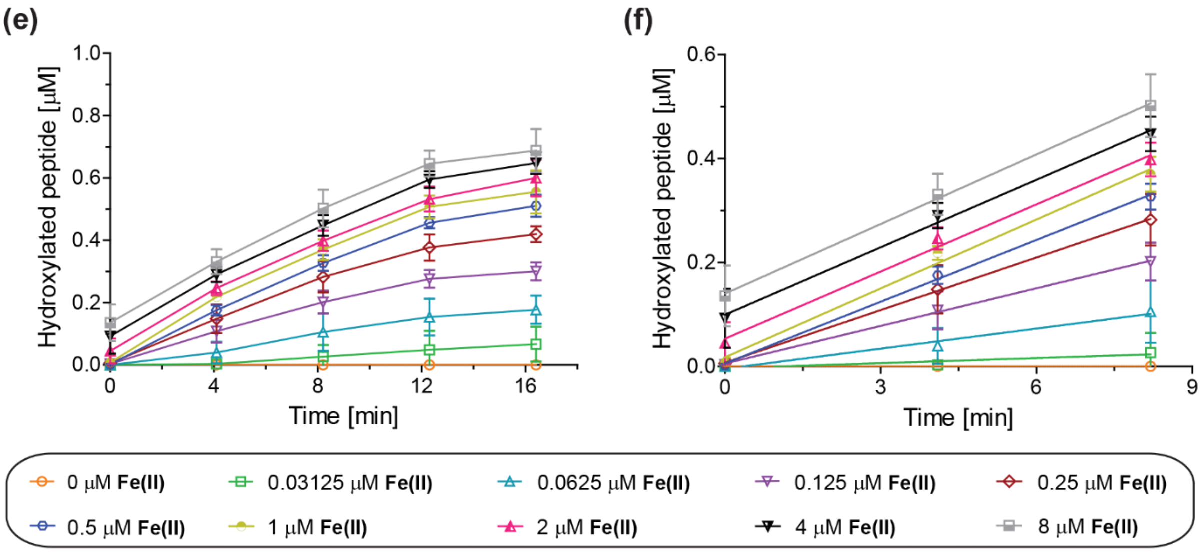

(**g**) Time course of the JMJD5-catalyzed hydroxylation reaction of the $RPS6_{128\text{-}148}$ peptide for the shown concentrations of Fe(II) in the absence of L-ascorbic acid using $RPS6_{128\text{-}148}$ (4.0 µM) and 2OG (20 µM); and (**h**) hydroxylation rates used to determine kinetic parameters of JMJD5 for Fe(II) in the absence of L-ascorbic acid.

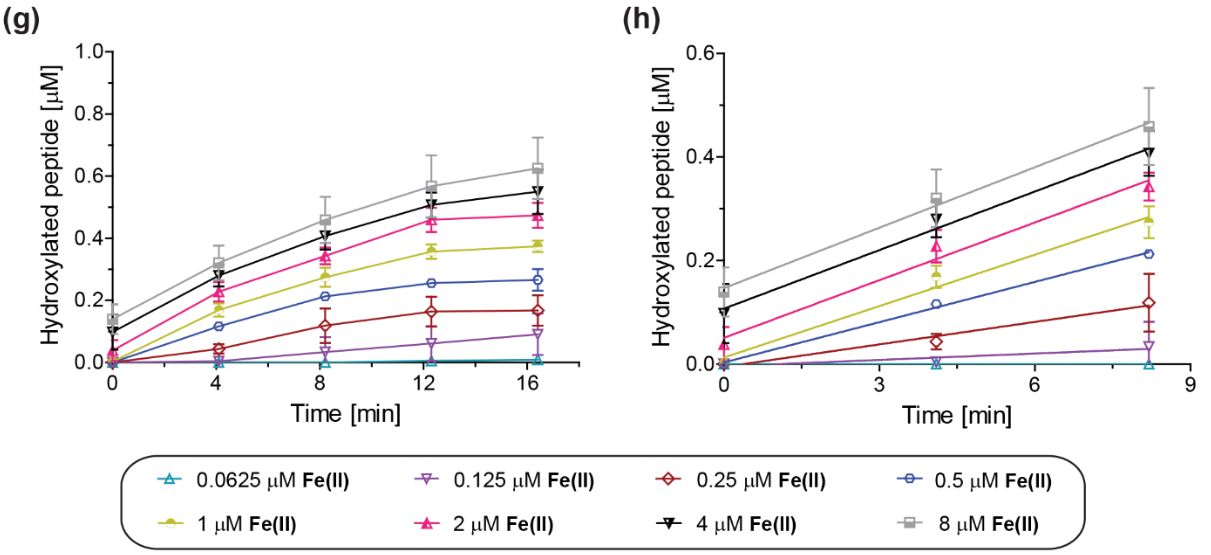

(**i**) Time course of the JMJD5-catalyzed hydroxylation reaction of the $RPS6_{128\text{-}148}$ peptide for the shown concentrations of $RPS6_{128\text{-}148}$ using L-ascorbic acid (100 µM), ammonium iron(II) sulfate hexahydrate (20 µM, $(NH_4)_2Fe(SO_4)_2{\cdot}6H_2O$), and 2OG (50 µM); and (**j**) hydroxylation rates used to determine kinetic parameters of JMJD5 for $RPS6_{128\text{-}148}$.

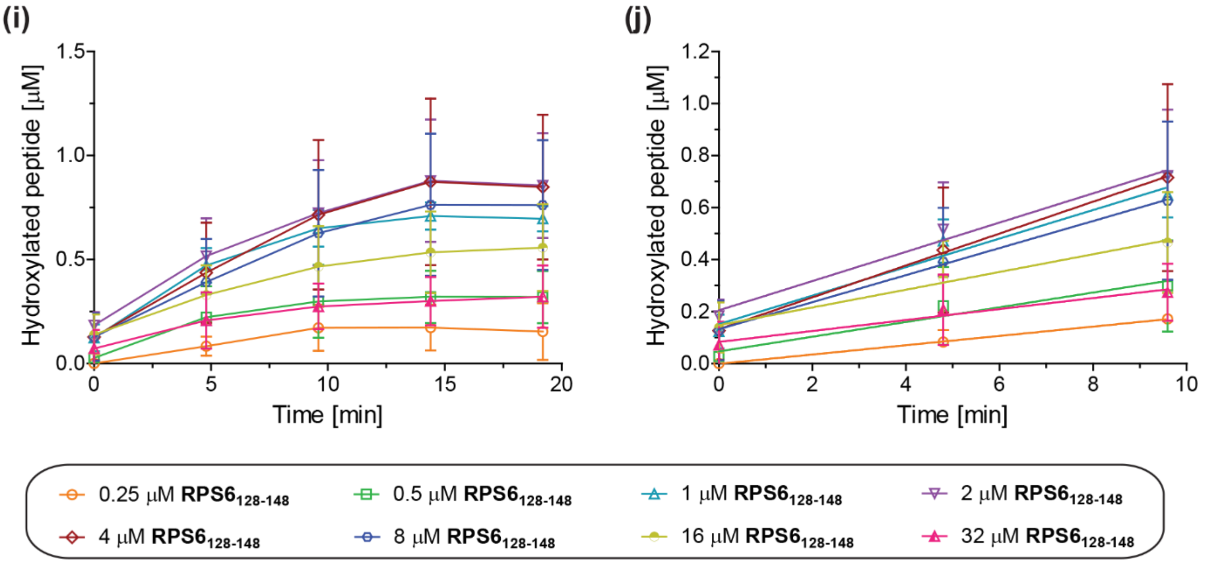

Supplementary Figure S2. Rates of JMJD5-catalyzed $RPS6_{128-148}$ hydroxylations used to determine kinetic parameters for (1*R*)-3-(carboxycarbonyl)cyclopentane-1-carboxylic acid. Maximum velocities (v_{max}^{app}) and Michaelis constants (K_m^{app}) of JMJD5 were determined in independent triplicates for (1*R*)-3-(carboxycarbonyl)cyclopentane-1-carboxylic acid (**14**), monitoring the JMJD5-catalyzed hydroxylation of the $RPS6_{128-148}$ peptide (TVPRRLGPKRASRIRKLFNLS; JMJD5 catalyses the hydroxylation of R137[1]) by SPE-MS as described (Experimental Section). Conditions: JMJD5 (0.15 µM), $RPS6_{128-148}$ peptide (4.0 µM), L-ascorbic acid (100 µM), and ammonium iron(II) sulfate hexahydrate (20 µM, $(NH_4)_2Fe(SO_4)_2 \cdot 6H_2O$) in buffer (50 mM MOPS, pH 7.5, 20 °C). Measurement times were normalized to the first sample injection analyzed after the addition of JMJD5 to the Substrate Mixture (t = 0 s), by which time low levels of $RPS6_{128-148}$ hydroxylation were manifest. Data are shown as the mean of three independent runs (n = 3; mean ± SD).

(**a**) Time course of the JMJD5-catalyzed hydroxylation reaction of the $RPS6_{128-148}$ peptide for the shown concentrations of (1*R*)-3-(carboxycarbonyl)cyclopentane-1-carboxylic acid (**14**); and (**b**) hydroxylation rates used to determine kinetic parameters of JMJD5 for **14**.

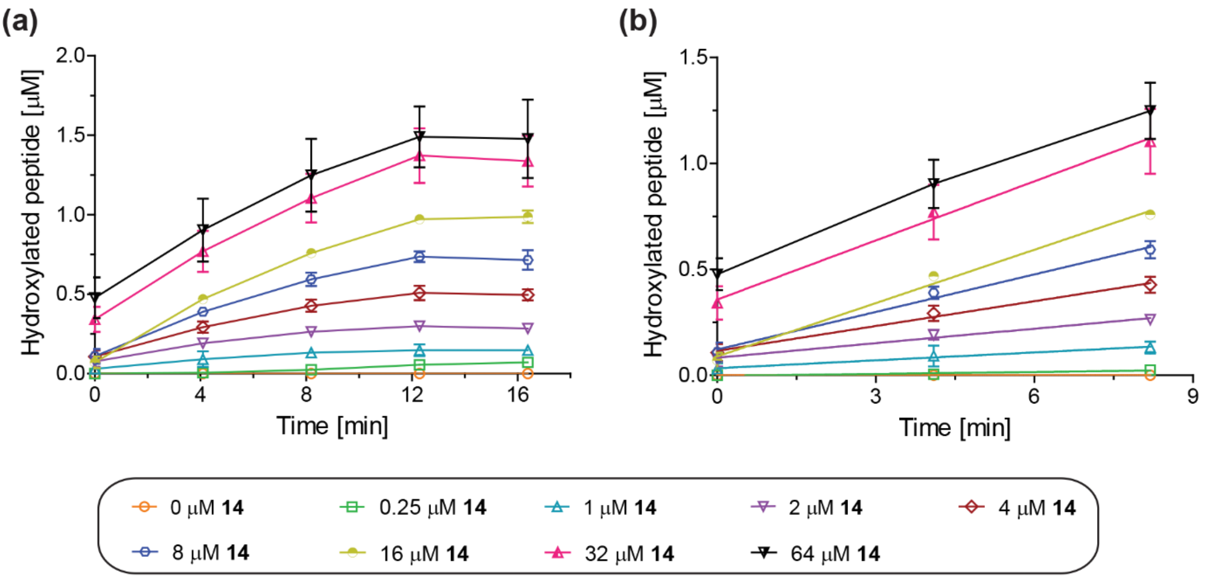

Supplementary Figure S3. Rates of KDM4E-catalyzed N^{ε}-trimethyl lysine demethylations of a histone 3-derived peptide used to determine kinetic parameters for 2OG. Maximum velocities (v_{max}^{app}) and Michaelis constants (K_{m}^{app}) of KDM4E were determined in independent triplicates for 2OG, monitoring the KDM4E-catalyzed N^{ε}-trimethyl lysine demethylation of the histone 3 K9(me3)-derived peptide ARTAQTARK(me3)STGGIA[2] by SPE-MS as described;[3] note that mono-, di-, and tri-demethylated product peptides were observed. Conditions: KDM4E (0.15 µM), ARTAQTARK(me3)STGGIA substrate peptide (10.0 µM)[2], L-ascorbic acid (100 µM), and ammonium iron(II) sulfate hexahydrate (20 µM, $(NH_4)_2Fe(SO_4)_2 \cdot 6H_2O$) in buffer (50 mM MES, pH 7.0, 20 °C). Measurement times were normalized to the first sample injection analyzed after the addition of KDM4E to the Substrate Mixture (t = 0 s), by which time low levels of N^{ε}-trimethyl lysine demethylation were manifest. Data are shown as the mean of three independent runs (n = 3; mean ± SD).

(**a**) Time course of the KDM4E-catalyzed N^{ε}-trimethyl lysine demethylation reaction of the ARTAQTARK(me3)STGGIA peptide[2] for the shown concentrations of 2OG; (**b**) N^{ε}-trimethyl lysine demethylation rates used to determine kinetic parameters of KDM4E for 2OG; (**c**) determination of the KDM4E v_{max}^{app} and K_{m}^{app} values for 2OG (*i.e.* 20.6 ± 1.3 nM·s^{-1} and 4.2 ± 1.1 µM, respectively).

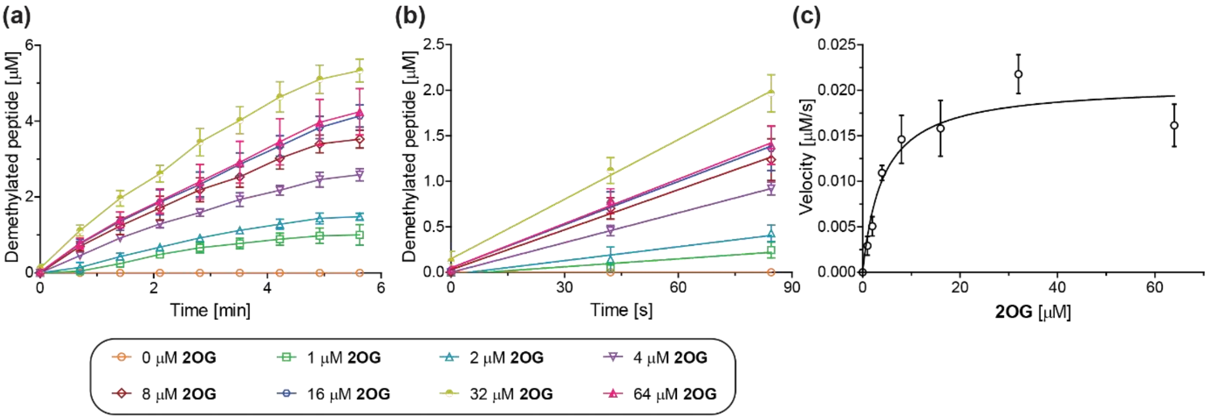

Supplementary Figure S4. Docking studies indicate that both (1*R*,3*S*)-14 and (1*R*,3*R*)-14 may bind to the JMJD5 active site (continues on the following page). The reported JMJD5:Mn:pyridine-2,4-dicarboxylic acid (2,4-PDCA) crystal structure (PDB ID: 6I9L[4]) was prepared for docking studies by adding hydrogen atoms and by checking asparagine, glutamine, and histidine residues for flips with REDUCE,[5] using the MolProbity server.[6] The pKa values of all ionizable groups were calculated using PropKa[7] and protonated using Pymol (version 4.6.0) at pH 7.5. The active site Mn ion was substituted for a Fe(II) ion. Alternative side chain conformations, bound ligands, and all crystallographic waters, excluding the metal-bound water (residue ID: HOH230), were removed using Pymol to give the JMJD5:Fe complex. Coordinates of (1*R*,3*S*)-**14** and (1*R*,3*R*)-**14** were generated using Discovery Studio 2016.

Docking studies were performed using the protein-ligand docking software GOLD (version 5.1)[8] and the pre-prepared JMJD5:Fe complex. For both (1*R*,3*S*)- and (1*R*,3*R*)-**14**, 100 genetic algorithm (GA) runs were carried out, the ChemScore scoring function was used to evaluate the predicted binding poses. The binding site was defined as all atoms within 8 Å of the crystallographic binding pose of 2,4-PDCA; JMJD5 active site residues Y272, S318, and K336 were set as 'Flexible'. The 'Allow early termination' option was disabled and the 'Generate diverse solutions' option was enabled. All other settings were used as the default.

(**a**-**b**) Views of the predicted interactions of (**a**) (1*R*,3*R*)-**14** and (**b**) (1*R*,3*S*)-**14** with the JMJD5:Fe complex, which was computationally prepared as described above. The distal carboxylate of 2OG derivative **14** is positioned to interact with the side chains of Y272, K336, and S410 in both the predicted JMJD5:Fe:(1*R*,3*R*)-**14** and the predicted JMJD5:Fe:(1*R*,3*S*)-**14** complex, in a manner similar to that of the C5 carboxylate of 2OG in the reported JMJD5:Mn:2OG crystal structure (PDB ID: 6F4N[1], panel **e**); (**c**-**d**) views of the predicted interactions of (**c**) (1*R*,3*R*)-**14** and (**d**) (1*R*,3*S*)-**14** with the JMJD5:Fe complex, which was computationally prepared as described above. The distal carboxylate of 2OG derivative **14** is positioned to either interact with the side chains of K336 and Ser410 in the predicted JMJD5:Fe:(1*R*,3*R*)-**14** complex or with the side chains of Y272 and K336 in the predicted JMJD5:Fe:(1*R*,3*S*)-**14** complex, a binding mode which differs from that of the C5 carboxylate of 2OG in the reported JMJD5:Mn:2OG crystal structure (PDB ID: 6F4N[1], panel **e**). The docking results indicate that both (1*R*,3*S*)-**14** and (1*R*,3*R*)-**14** may bind to the 2OG binding pocket at the JMJD5 active site, which is consistent with the observed JMJD5-catalysed hydroxylation of $RPS6_{128-148}$ in the presence of **14**. The calculated highest fitness scores of the two diastereomers were comparable (92.4 for (1*R*,3*S*)-**14**; 95.5 for (1*R*,3*R*)-**14**), suggesting that the docking studies do not indicate a preferential binding mode of **14**.

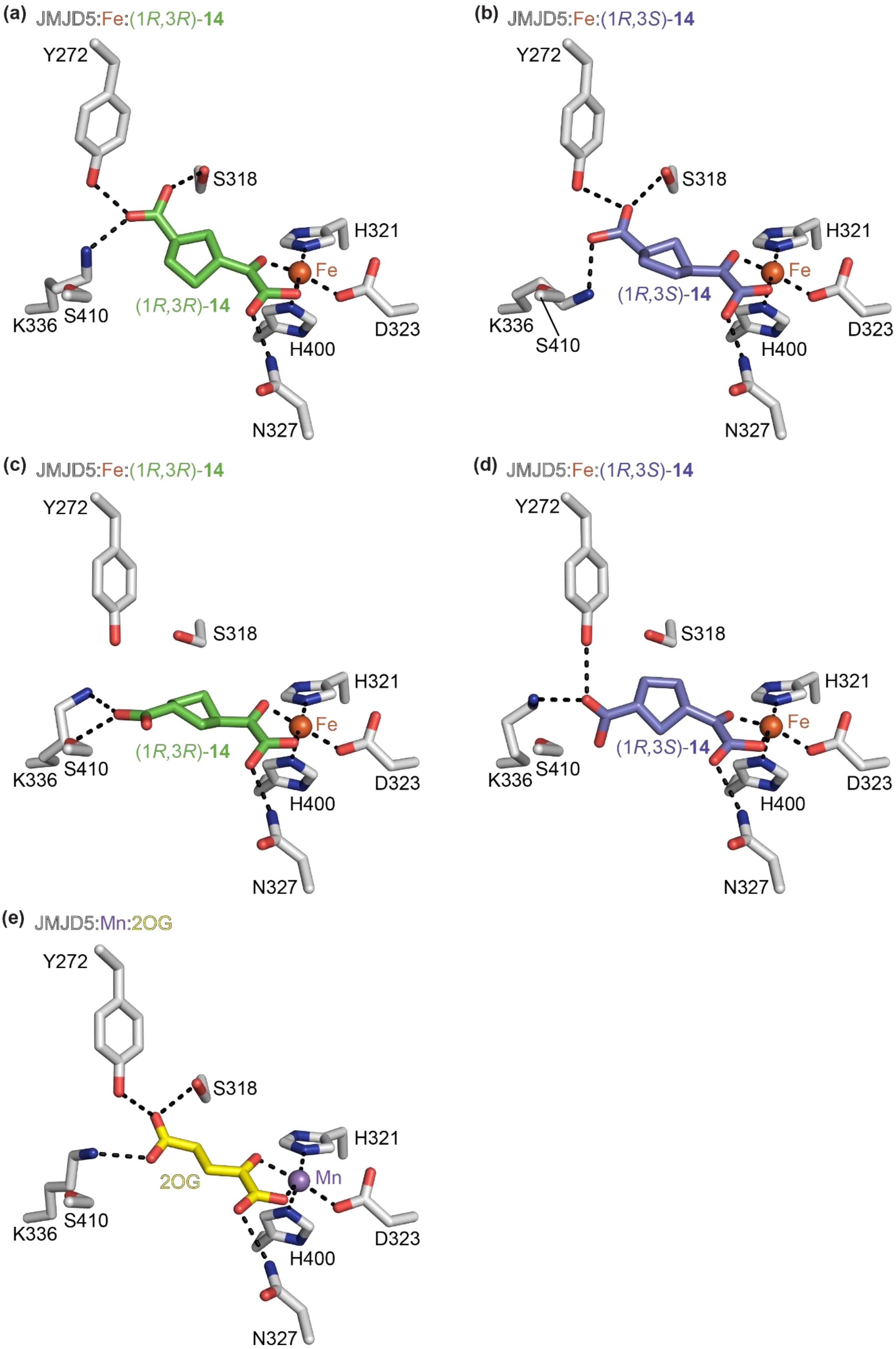
(a) JMJD5:Fe:(1R,3R)-14
Y272
S318
H321
Fe
(1R,3R)-14
K336
S410
D323
H400
N327
(b) JMJD5:Fe:(1R,3S)-14
Y272
S318
H321
Fe
(1R,3S)-14
K336
S410
D323
H400
N327
(c) JMJD5:Fe:(1R,3R)-14
Y272
S318
H321
Fe
(1R,3R)-14
K336
S410
D323
H400
N327
(d) JMJD5:Fe:(1R,3S)-14
Y272
S318
H321
Fe
(1R,3S)-14
K336
S410
D323
H400
N327
(e) JMJD5:Mn:2OG
Y272
S318
H321
Mn
2OG
K336
S410
D323
H400
N327

2. Supplementary tables

Supplementary Table S1. 2OG oxygenases react selectively with 2OG derivatives (continues on the following page). SPE-MS turnover assays to investigate the effect of 2OG derivatives on isolated recombinant human JMJD5 and KDM4E were performed in the absence of 2OG as described (Experimental Section). The results were compared to those reported for isolated recombinant human FIH and AspH,[9-10] which were obtained using SPE-MS that employed similar conditions to those of the JMJD5 and KDM4E assays.

The results reveal that the ability of the tested human 2OG oxygenases to accept cosubstrates other than 2OG varies substantially. AspH can employ 11 of the tested 34 2OG derivatives as cosubstrates, whereas both JMJD5 and FIH can accept 6 2OG derivatives as substrates and KDM4E only 3 2OG derivatives. Note, however, that at least the ability of FIH to react with 2OG derivatives is, to some extent, substrate dependent.[10]

The results also reveal the potential of 2OG derivatives to selectively react with specific 2OG oxygenases. For example, some 2OG derivatives which bear an aromatic scaffold, *i.e.* **17**, **28-32**, and **34**, are cosubstrates of AspH, but not of JMJD5, FIH, and KDM4E. Similarly, 4-ethyl-2OG (**10**) and 4-propyl-2OG (**11**) are cosubstrates for JMJD5 and FIH, but not for KDM4E and AspH (Entries 14 and 15), in accord with the reported structural similarities of the JMJD5 and FIH active sites.[11-12]

	[a]**2OG derivative**	[b]**JMJD5**	[c]**KDM4E**	[d]**FIH**[10]	[e]**AspH**[9]		[a]**2OG derivative**	[b]**JMJD5**	[c]**KDM4E**	[d]**FIH**[10]	[e]**AspH**[9]
1	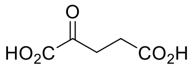 **2OG**	~40%	~70%	~50%	>95%	8	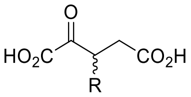 **5**: R = $CH_2(4\text{-}F_3COC_6H_4)$	<1%	<1%	<1%	<1%
2	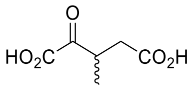 **1**	~10%	~10%	~55%	>95%	9	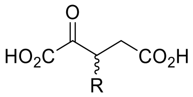 **6**: R = $CH_2(4\text{-}MeOC_6H_4)$	~10%	~5%	<1%	<1%
3	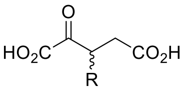 **2**: R = CH_2CH_3	<1%	<1%	~2%	<1%	10	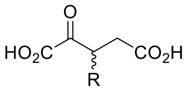 **20**: R = $CH_2(3,5\text{-}Me_2C_6H_4)$	<1%	<1%	<1%	<1%
4	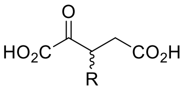 **3**: R = $CH_2CH_2CH_3$	<1%	<1%	<1%	<1%	11	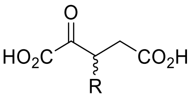 **7**: R = CH_2(9,9-dimethyl-9*H*-fluoren-2-yl)	<1%	<1%	<1%	<1%
5	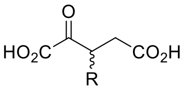 **18**: R = $CH_2CH_2C(CH_3)_3$	<1%	<1%	<1%	<1%	12	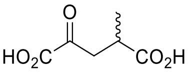 **8**	~10%	<1%	~45%	~10%
6	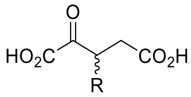 **4**: R = $CH_2CH_2CH_2Ph$	<1%	~2%	<1%	<1%	13	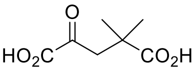 **9**	<1%	<1%	<5%	<1%
7	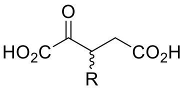 **19**: R = $CH_2(4\text{-}FC_6H_4)$	<1%	<1%	<1%	<1%	14	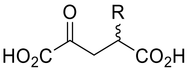 **10**: R = CH_2CH_3	~10%	<1%	~10%	<1%

	[a]2OG derivative	[b]JMJD5	[c]KDM4E	[d]FIH[10]	[e]AspH[9]		[a]2OG derivative	[b]JMJD5	[c]KDM4E	[d]FIH[10]	[e]AspH[9]
15	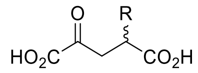 **11**: R = $CH_2CH_2CH_3$	~10%	<1%	~5%	<1%	26	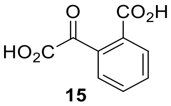 **15**	<1%	<1%	<1%	<1%
16	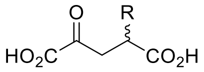 **21**: R = $CH_2CH(CH_3)_2$	<1%	<1%	<1%	<1%	27	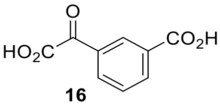 **16**	<1%	<1%	<1%	<1%
17	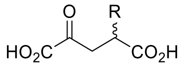 **22**: R = $CH_2CH_2C(CH_3)_3$	<1%	<1%	<1%	<1%	28	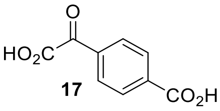 **17**	<1%	<1%	<1%	~80%
18	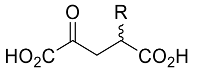 **12**: R = $CH_2CH_2CH_2Ph$	<1%	<1%	<1%	<1%	29	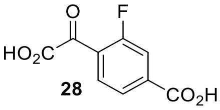 **28**	<1%	<1%	<1%	~45%
19	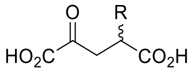 **13**: R = CH_2Ph	<1%	<1%	<1%	<1%	30	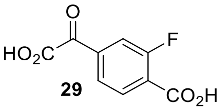 **29**	<1%	<1%	<1%	~8%
20	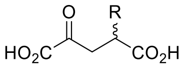 **23**: R = CH_2(2-naphthyl)	<1%	<1%	<1%	<1%	31	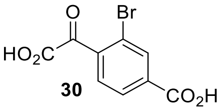 **30**	<1%	<1%	<1%	~20%
[f]21	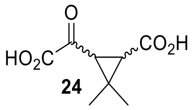 **24**	<1%	<1%	<1%	<1%	32	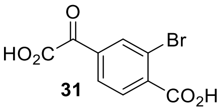 **31**	<1%	<1%	<1%	~8%
[g]22	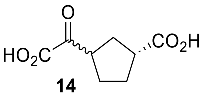 **14**	~35%	<1%	~2%	~15%	33	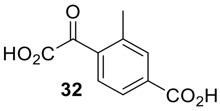 **32**	<1%	<1%	<1%	~5%
23	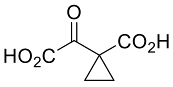 **25**	<1%	<1%	<1%	<1%	34	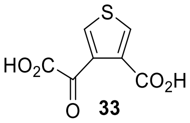 **33**	<1%	<1%	<1%	<1%
[h]24	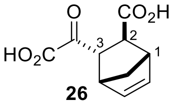 **26**	<1%	<1%	<1%	<1%	35	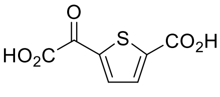 **34**	<1%	<1%	<1%	~15%
25	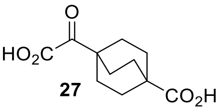 **27**	<1%	<1%	<1%	~8%						

a) Chiral 2OG derivatives were prepared as racemic mixtures as reported;[9-10] b) JMJD5 (0.15 μM), 2OG derivative (400 μM), Fe(II) (20 μM), and $RPS6_{128-148}$ (2.0 μM) in buffer (50 mM MOPS, pH 7.5); c) KDM4E (0.15 μM), 2OG derivative (330 μM), Fe(II) (50 μM), and ARTAQTARK(me3)STGGIA (a histone 3 K9(me3) derivative)[2] (10.0 μM) in buffer (50 mM MES, pH 7.0); d) reported using: FIH (0.15 μM), 2OG derivative (330 μM), Fe(II) (50 μM), and HIF-$1\alpha_{788-822}$[13] (5.0 μM) in buffer (50 mM Tris, 50 mM NaCl, pH 7.5);[10] e) reported using: AspH (0.1 μM), 2OG derivative (330 μM), Fe(II) (50 μM), and $hFX\text{-}CP_{101-119}$ (2.0 μM) in buffer (50 mM HEPES, pH 7.5);[9] f) mixture of racemic diastereomers, dr (*cis*:*trans*) = 2.5:1; g) mixture of diastereomers, dr (*cis*:*trans*) = 1:1; h) (±)-(*2-exo,3-endo*)-diastereomer.

Supplementary Table S2. 2OG derivatives have potential to selectively inhibit 2OG oxygenases (continues on the following two pages). SPE-MS JMJD5 inhibition assays were performed as described (Experimental Section) employing JMJD5 (0.15 µM), 2OG (2.0 µM), Fe(II) (2.0 µM), and $RPS6_{128-148}$ (2.0 µM), and LAA (100 µM) in buffer (50 mM MOPS, pH 7.5, 20 °C). SPE-MS KDM4E inhibition assays were performed as reported.[3] The inhibition of JMJD5 and KDM4E by 2OG derivatives was compared to reported results for AspH[9] and FIH[10]. The reported broad-spectrum 2OG oxygenase inhibitor pyridine-2,4-dicarboxylic acid (2,4-PDCA)[14] was used as a positive inhibition control (Entry 1). None of the 34 2OG derivatives investigated for JMJD5 inhibition were efficient inhibitors of isolated recombinant human JMJD5, whereas 17 and 23 of them have been reported to inhibit isolated recombinant human FIH and AspH, respectively.[9-10] The lack of efficient inhibition of JMJD5 by C3 and/or C4 substituted 2OG derivatives may reflect the crystallographic observation that the side chains of JMJD5 Trp310, Leu329, and Val402 form a relatively tight hydrophobic pocket around the 2OG ethylene unit, potentially resulting in a reduced ability to accommodate sterically bulky substituents at the 2OG C3 and C4 positions.[1]

Interestingly, five 2OG derivatives inhibited KDM4E catalysis, *i.e.* **7**, **8**, **12**, **14**, and **23**, with **7**, **8**, and **23** being most potent (IC_{50}s ~ 6-7 µM, Entries 11, 12, and 20). Note that both 2OG derivatives **7** and **23** also efficiently inhibit AspH and FIH.[9-10] Interestingly, 4-methyl-2OG (**8**) appears to selectively inhibit KDM4E over JMJD5, AspH, and FIH (Entry 12); however, **8** is a substrate for JMJD5, AspH, and FIH which might limit its utility as a KDM4E inhibitor (Supplementary Table S1).

The results reveal potential for selective inhibition of AspH over FIH, JMJD5, and KDM4E, *i.e.* 2OG derivatives **9**, **25**, **26**, and **34** appear to only inhibit AspH under the tested conditions (Entries 13, 23, 24, and 35). Of these selective AspH inhibitors, 4,4-dimethyl-2OG (**13**) is the most efficient AspH inhibitor (IC_{50} ~ 0.3 µM, Entry 13).

	[a]2OG derivative	[b]IC_{50} JMJD5 [µM]	[c]IC_{50} KDM4E [µM]	[d]IC_{50} FIH [µM][10]	[e]IC_{50} AspH [µM][9]
1	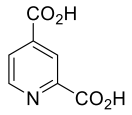 **2,4-PDCA**	0.33 ± 0.07	0.12 ± 0.05	4.7 ± 1.6[3]	0.03 ± 0.01[3]
2	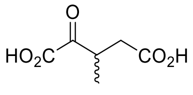 **1**	>50	>30	>50	>50
3	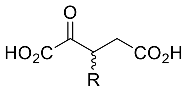 **2**: R = CH_2CH_3	>20	>50	4.3 ± 0.1	1.2 ± 0.5
4	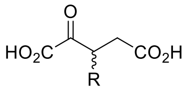 **3**: R = $CH_2CH_2CH_3$	>30	>50	2.7 ± 0.1	5.7 ± 1.1
5	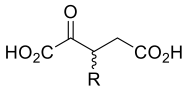 **18**: R = $CH_2CH_2C(CH_3)_3$	>30	>50	31.0 ± 0.1	48.2 ± 13.1
6	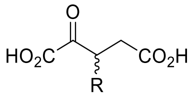 **4**: R = $CH_2CH_2CH_2Ph$	>50	>50	5.7 ± 0.6	6.8 ± 0.9
7	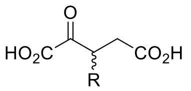 **19**: R = $CH_2(4\text{-}FC_6H_4)$	>50	>50	7.5 ± 0.7	2.6 ± 0.8
8	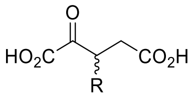 **5**: R = $CH_2(4\text{-}F_3COC_6H_4)$	>50	>50	12.7 ± 1.3	6.3 ± 2.6
9	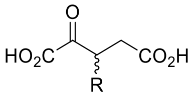 **6**: R = $CH_2(4\text{-}MeOC_6H_4)$	>50	>50	3.6 ± 0.3	3.6 ± 1.4
10	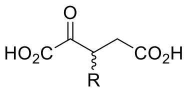 **20**: R = $CH_2(3,5\text{-}Me_2C_6H_4)$	>50	>50	>50	4.7 ± 0.1

	[a]2OG derivative	[b]IC50 JMJD5 [μM]	[c]IC50 KDM4E [μM]	[d]IC50 FIH [μM][10]	[e]IC50 AspH [μM][9]		[a]2OG derivative	[b]IC50 JMJD5 [μM]	[c]IC50 KDM4E [μM]	[d]IC50 FIH [μM][10]	[e]IC50 AspH [μM][9]
11	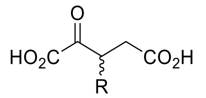 **7**: R = CH_2(9,9-dimethyl-9*H*-fluoren-2-yl)	>50	6.1 ± 0.3	1.9 ± 0.1	4.3 ± 3.8	[h]24	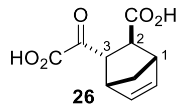 **26**	>50	>50	>50	19.3 ± 1.6
12	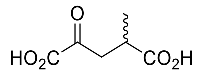 **8**	>50	6.6 ± 2.5	>50	>50	25	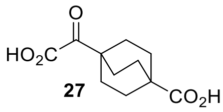 **27**	>50	>50	>50	>50
13	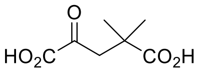 **9**	>50	>30	>50	0.31 ± 0.10	26	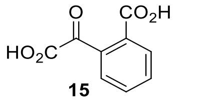 **15**	>50	>50	>50	>50
14	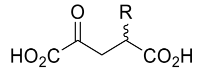 **10**: R = CH_2CH_3	>50	>50	19.3 ± 7.7	0.61 ± 0.09	27	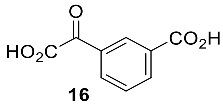 **16**	>50	>50	>50	>50
15	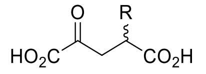 **11**: R = $CH_2CH_2CH_3$	>50	>50	14.8 ± 0.7	0.47 ± 0.08	28	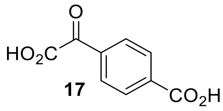 **17**	>50	>50	>50	>50
16	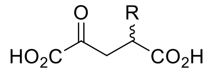 **21**: R = $CH_2CH(CH_3)_2$	>50	>50	35.3 ± 2.0	0.51 ± 0.12	29	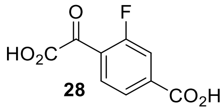 **28**	>50	>50	>50	>50
17	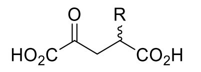 **22**: R = $CH_2CH_2C(CH_3)_3$	>50	>50	38.9 ± 2.1	0.70 ± 0.11	30	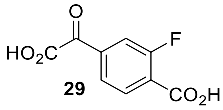 **29**	>50	>50	>50	>50
18	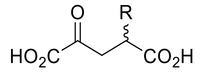 **12**: R = $CH_2CH_2CH_2Ph$	>50	24.0 ± 5.1	0.9 ± 0.1	0.25 ± 0.05	31	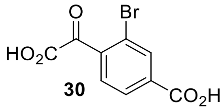 **30**	>50	>50	>50	>50
19	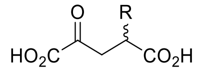 **13**: R = CH_2Ph	>50	>50	3.6 ± 0.2	0.43 ± 0.05	32	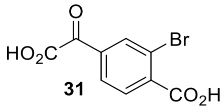 **31**	>50	>50	>50	>50
20	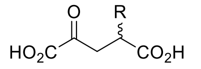 **23**: R = CH_2(2-naphthyl)	>50	6.9 ± 1.4	2.5 ± 0.1	0.17 ± 0.03	33	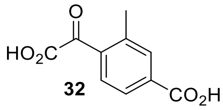 **32**	>50	>50	>50	>50
[f]21	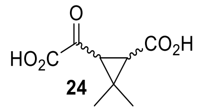 **24**	>50	>50	19.4 ± 0.6	5.2 ± 1.7	34	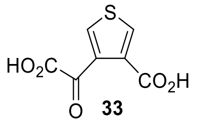 **33**	>50	>50	>50	>50
[g]22	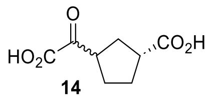 **14**	>50	27.9 ± 3.5	>50	5.0 ± 1.2	35	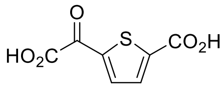 **34**	>30	>50	>50	12.9 ± 1.3
23	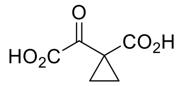 **25**	>50	>50	>50	3.3 ± 1.0						

a) Chiral 2OG derivatives were prepared as racemic mixtures as reported;[9-10] b) JMJD5 (0.15 μM), $RPS6_{128-148}$ (2.0 μM), LAA (100 μM), Fe(II) (2.0 μM), and 2OG (2.0 μM) in buffer (50 mM MOPS, pH 7.5, 20 °C); c) KDM4E (0.15 μM), ARTAQTARK(me3)STGGIA (a histone 3 K9(me3) derivative)[2] (10.0 μM), LAA (100 μM), FAS (10 μM), and 2OG (10 μM) in buffer (50 mM MES, pH 7.0, 20 °C); d) reported using: FIH (0.15 μM), HIF-$1\alpha_{788-822}$[13] (5.0 μM), LAA (100 μM), FAS (10 μM), and 2OG (10 μM) in buffer (50 mM Tris, 50 mM NaCl, pH 7.5, 20 °C);[10] e) reported using: AspH (0.05 μM), substrate peptide (hFX-$CP_{101-119}$;[15-16] 1.0 μM), LAA (100 μM), FAS (2.0 μM), and 2OG (3.0 μM) in buffer (50 mM HEPES, pH 7.5, 20 °C);[9] f) mixture of racemic diastereomers, dr (*cis*:*trans*) = 2.5:1; g) mixture of diastereomers, dr (*cis*:*trans*) = 1:1; h) (±)-(*2-exo,3-endo*)-diastereomer.

3. References

1. S. E. Wilkins, M. S. Islam, J. M. Gannon, S. Markolovic, R. J. Hopkinson, W. Ge, C. J. Schofield and R. Chowdhury, *Nat. Commun.*, 2018, **9**, 1180.
2. S. E. Hutchinson, M. V. Leveridge, M. L. Heathcote, P. Francis, L. Williams, M. Gee, J. Munoz-Muriedas, B. Leavens, A. Shillings, E. Jones, P. Homes, S. Baddeley, C.-w. Chung, A. Bridges and A. Argyrou, *J. Biomol. Screen.*, 2012, **17**, 39-48.
3. L. Brewitz, Y. Nakashima, A. Tumber, E. Salah and C. J. Schofield, *J. Fluor. Chem.*, 2021, **247**, 109804.
4. M. S. Islam, M. Markoulides, R. Chowdhury and C. J. Schofield, *Sci. Rep.*, 2022, **12**, 20680.
5. J. M. Word, S. C. Lovell, J. S. Richardson and D. C. Richardson, *J. Mol. Biol.*, 1999, **285**, 1735-1747.
6. I. W. Davis, A. Leaver-Fay, V. B. Chen, J. N. Block, G. J. Kapral, X. Wang, L. W. Murray, W. B. Arendall III, J. Snoeyink, J. S. Richardson and D. C. Richardson, *Nucleic Acids Res.*, 2007, **35**, W375-W383.
7. C. R. Søndergaard, M. H. M. Olsson, M. Rostkowski and J. H. Jensen, *J. Chem. Theory Comput.*, 2011, **7**, 2284-2295.
8. G. Jones, P. Willett, R. C. Glen, A. R. Leach and R. Taylor, *J. Mol. Biol.*, 1997, **267**, 727-748.
9. L. Brewitz, Y. Nakashima and C. J. Schofield, *Chem. Sci.*, 2021, **12**, 1327-1342.
10. Y. Nakashima, L. Brewitz, A. Tumber, E. Salah and C. J. Schofield, *Nat. Commun.*, 2021, **12**, 6478.
11. H. Wang, X. Zhou, M. Wu, C. Wang, X. Zhang, Y. Tao, N. Chen and J. Zang, *Acta Cryst. D*, 2013, **69**, 1911-1920.
12. P. A. Del Rizzo, S. Krishnan and R. C. Trievel, *Mol. Cell. Biol.*, 2012, **32**, 4044-4052.
13. P. Koivunen, M. Hirsilä, V. Günzler, K. I. Kivirikko and J. Myllyharju, *J. Biol. Chem.*, 2004, **279**, 9899-9904.
14. N. R. Rose, M. A. McDonough, O. N. F. King, A. Kawamura and C. J. Schofield, *Chem. Soc. Rev.*, 2011, **40**, 4364-4397.
15. I. Pfeffer, L. Brewitz, T. Krojer, S. A. Jensen, G. T. Kochan, N. J. Kershaw, K. S. Hewitson, L. A. McNeill, H. Kramer, M. Münzel, R. J. Hopkinson, U. Oppermann, P. A. Handford, M. A. McDonough and C. J. Schofield, *Nat. Commun.*, 2019, **10**, 4910.
16. L. Brewitz, A. Tumber, I. Pfeffer, M. A. McDonough and C. J. Schofield, *Sci. Rep.*, 2020, **10**, 8650.
